# Thiol-ene-based microfluidic chips for glycopeptide enrichment and online digestion of inflammation-related proteins osteopontin and immunoglobulin G

**DOI:** 10.1007/s00216-022-04498-2

**Published:** 2023-01-06

**Authors:** Yuye Zhou, Alexander Jönsson, Drago Sticker, Guojun Zhou, Zishuo Yuan, Jörg P. Kutter, Åsa Emmer

**Affiliations:** 1 Department of Chemistry, Analytical Chemistry, School of Engineering Sciences in Chemistry, Biotechnology and Health, KTH Royal Institute of Technology, 100 44 Stockholm, Sweden; 2 Department of Health Technology, Technical University of Denmark, 2800 Kongens Lyngby, Denmark; 3 Novo Nordisk A/S, Biophysics and Formulation, 2760 Måløv, Denmark; 4 Department of Materials and Environmental Chemistry, Stockholm University, 106 91 Stockholm, Sweden; 5 Department of Pharmacy, University of Copenhagen, 2100 Copenhagen, Denmark

**Keywords:** Thiol-ene microchip, Protein digestion, Glycopeptide enrichment, Mass spectrometry

## Abstract

Proteins, and more specifically glycoproteins, have been widely used as biomarkers, e.g., to monitor disease states. Bottom-up approaches based on mass spectrometry (MS) are techniques commonly utilized in glycoproteomics, involving protein digestion and glycopeptide enrichment. Here, a dual function polymeric thiol-ene-based microfluidic chip (TE microchip) was applied for the analysis of the proteins osteopontin (OPN) and immunoglobulin G (IgG), which have important roles in autoimmune diseases, in inflammatory diseases, and in coronavirus disease 2019 (COVID-19). TE microchips with larger internal surface features immobilized with trypsin were successfully utilized for OPN digestion, providing rapid and efficient digestion with a residence time of a few seconds. Furthermore, TE microchips surface-modified with ascorbic acid linker (TEA microchip) have been successfully utilized for IgG glycopeptide enrichment. To illustrate the use of the chips for more complex samples, they were applied to enrich IgG glycopeptides from human serum samples with antibodies against severe acute respiratory syndrome coronavirus 2 (SARS-CoV-2). The dual functional TE microchips could provide high throughput for online protein digestion and glycopeptide enrichment, showing great promise for future extended applications in proteomics and the study of related diseases.

**Graphical Abstract:**

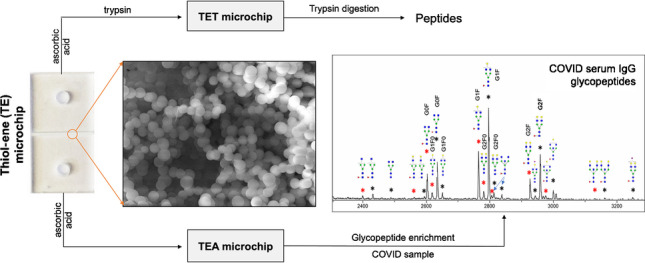

**Supplementary Information:**

The online version contains supplementary material available at 10.1007/s00216-022-04498-2.

## Introduction

Proteins are of high importance in life science research, and in the analysis of biological samples, due to their wide range of functions in the body [1, 2]. Interactions between proteins or between proteins and other molecules (e.g., DNA, RNA) can lead to changes in the proteins and can cause diseases [3]. Post-translational modifications (PTMs) of proteins control many biological processes and broaden the functionality of proteins. One of the most common types of PTMs, glycosylation, has been widely reported as associated with diseases, such as cardiometabolic disorders, cancers, autoimmune diseases and inflammatory diseases, and in coronavirus disease 2019 (COVID-19) [4–8]. Due to the central role of proteins in biological functions and control mechanisms associated with diseases, proteomics, the large-scale study of proteins, is especially promising in research regarding biomarkers [9].

The study of proteins has been focused on amino acid sequence determination, abundance determination, and PTM localization and identification [10]. Bottom-up approaches based on mass spectrometry (MS), including electrospray ionization mass spectrometry (ESI–MS) and matrix-assisted laser desorption/ionization-time of flight mass spectrometry (MALDI-TOF–MS), and tandem MS are techniques widely used for the task [11–13]. Protein digestion using a proteolytic enzyme is an unavoidable step involved in these methods, facilitating the identification through analysis of smaller peptides. In order to reduce the sample volume needed for the analysis of biological samples, and to increase the time-to-result efficiency, microfluidic devices with immobilized enzymes providing online digestion have been developed [14–25]. Microfluidic devices with integrated electrospray emitters, or prepared in fused silica capillary, or similar microfabricated devices could also be coupled to ESI–MS for online detection [23, 24, 26–29]. In glycosylation studies, the challenges, such as the ion suppression from non-glycopeptides and/or salts, and low ionization efficiency of glycopeptides in MS, make the direct detection of glycopeptides in a complex sample mixture difficult [30]. Therefore, methods for enrichment of glycopeptides from a complex biological sample matrix are crucial prior to MS detection and identification.

Here, a previously described microfluidic chip design with a high surface internal monolith (all features made from thiol-ene polymers) was applied for the analysis of inflammation-associated proteins osteopontin (OPN) and immunoglobulin G (IgG). OPN is a multifunctional protein, secreted and expressed in various tissues and cells, and detected in blood, urine, milk, and seminal fluid [31]. OPN is regarded as a proinflammatory cytokine, linked to a variety of acute and chronic inflammatory conditions, autoimmune diseases, cancers, and neurodegenerative diseases [31, 32]. Elevated levels of plasma OPN have been reported in, e.g., multiple sclerosis, Parkinson’s disease, Alzheimer’s disease, cancers, and coronary artery disease [32–36]. OPN has also been suggested to be a biomarker in COVID-19 due to its elevated levels in serum/plasma of COVID-19 severe patients [37–40]. IgG, the most abundant antibody in the human body, is also highly associated with chronic inflammatory and autoimmune diseases, such as rheumatoid arthritis, inflammatory bowel disease, Alzheimer’s disease, and complex regional pain syndrome [41–45]. The biological activity of IgG could be modulated by glycosylation, and the abundance of different glycoforms can change under certain physiological conditions, which make IgG glycosylation important in the study of chronic inflammatory and autoimmune diseases [41, 42]. In COVID-19, patients with severe symptoms have been characterized with high inflammatory cytokines and aberrations in IgG glycosylation, such as fucosylation and galactosylation [6–8]. Considering the role of IgG and OPN in autoimmunity and inflammation processes, these proteins are of specific concern.

In the present work, thiol-ene (TE) microchips with high surface monoliths were utilized for protein digestion and glycopeptide enrichment. As reported earlier, a TE microchip with immobilized pepsin was used for protein digestion [15]. Here, TE microchips with immobilized trypsin (TET microchips) were investigated for OPN digestion combined with MALDI-TOF–MS analysis. Digestion capacity, efficiency, sensitivity, and repeatability were investigated for recombinant human OPN (rhOPN) and compared with a conventional trypsin digestion method. Furthermore, the TE microchip was applied for online digestion and detection by the connection with ESI–MS. The rapid digestion and MS detection could be used in the future for fast quantification of OPN with the help of an internal standard. Moreover, ascorbic acid-modified TE microchips (TEA microchips) offered a hydrophilic surface in the monolith and were investigated for IgG glycopeptide enrichment, based on the hydrophilic interaction between glycopeptides and the adsorption material [13, 46, 47]. After optimization with IgG standard samples, TEA microchips were further assessed with human serum sample with antibodies against severe acute respiratory syndrome coronavirus 2 (SARS-CoV-2) and sample from healthy humans. This work aims to illustrate the potential for utilizing these and similar TE microchips for versatile and time-efficient applications in proteomics and the study of related diseases.

## Materials and methods

### Chemicals and consumables

Pentaerythritol-tetrakis(3-mercaptopropionate) (“tetrathiol”), triallyl-1,3,5-triazine-2,4,6-(1H,3H,5H)-trione (“triallyl”), 2-(boc amino) ethanethiol, and methanol (99.8%) were obtained from Sigma-Aldrich (Brøndby, Denmark). Lucirin TPO-L was obtained from BASF (Ludvigshafen, Germany). Acetonitrile (ACN), trifluoroacetic acid (TFA), DL-dithiothreitol (D9779, DTT), iodoacetamide (I670-9, IAA), immunoglobulin G (56,834-25MG, IgG, from human serum), trypsin (T1426, from bovine pancreas), and salts were purchased from Sigma-Aldrich (Stockholm, Sweden). L( +)-Ascorbic acid (99.0–100.5%) was from VWR chemicals (Stockholm, Sweden). Water (MQ H_2_O) was purified in a Millipore Synergy® 185 (Bedford, MA, USA) to a resistivity of 18.2 MΩ·cm at 25 °C. DEAE Affi-Gel® Blue Gel (DEAE) used for IgG extraction was obtained from Bio-RAD, Hercules, USA. Pierce® C18 Tips, 100 µL bed size, were from Thermo Fisher Scientific (Rockford, USA). Kinesis Tubing PEEK (TM) natural 1/32 inch × 0.015 inch connecting syringe and microchip was purchased from Fisher Scientific (Stockholm, Sweden). MALDI matrices 2,5-dihydroxybenzoic acid (DHB) and α-cyano-4-hydroxycinnamic acid (HCCA) and MALDI plate were obtained from Bruker Daltonics (Bremen, Germany).

### TE microchip preparation

TE microchips were prepared according to a previously described method [15]. Briefly, the TE microchips were prepared by pouring a stoichiometric mixture of tetrathiol and triallyl monomers into poly(dimethylsiloxane) molds and curing for 15 s on each side (top and bottom), under a UV flood light (160 mW cm^−2^ at 365 nm, Dymax EC 5000 Series UV curing flood lamp, Dymax Corp, Torrington, CT, USA). The TE microchips had a simple straight channel design with channel dimensions of 10 mm long × 200 μm deep × 400 μm wide and a channel volume of 0.8 μL. Fabricated microchips were placed in an oven at 60 °C overnight to dry and reach a stable weight and cooled down before further steps. An emulsion of off-stoichiometric thiol-ene (1:1.4 ratio tetrathiol/triallyl) in methanol (80% w/w) was mixed with a photoinitiator (10% v/v Lucirin TPO-L in ethanol, final concentration of 0.05% v/v in the emulsion mixture) and stirred for 1 min. The emulsion was directly injected into the TE microchip and immediately cured for 30 s under collimated UV light (20.5 mW cm^−2^ at 365 nm, LS-100-3C2 near UV light source, Bachur & Associates, Santa Clara, CA, USA) to form a “monolithic” structure providing a high internal surface area inside the microchannel (this feature is simply referred to as the “monolith” in the remainder of the manuscript).

### Characterization of TE microchips

The microchip was cut into a suitable size for scanning electron microscope (SEM) and Fourier transform infrared (FTIR) characterization. When characterized with SEM, samples were loaded onto the holder with the channel pointing straight upwards. SEM images were collected on JEOL JSM-7401F, a field emission SEM assembled with a field emission gun and Schottky emitter source. The images were recorded at an accelerating voltage of 2 kV or 5 kV for characterizing the surface of the monolith. For closer inspection of the porosity and the interlinked beads in the monolith, an accelerating voltage of 15 kV was applied. FTIR spectra were obtained using a Varian 670 FTIR.

### TE microchip linker and enzyme immobilization

Protected amino groups were introduced to the surface of the allyl-excess monolith by filling the chip with 2-(boc amino) ethanethiol containing 5% v/v photoinitiator (lucirin TPO-L). The reaction was performed under collimated UV light (30 s, 20.5 mW cm − 2 at 365 nm), according to the previously described method [15, 48]. A TE microchip with monolith was then fixed in a 3D-printed fluidic interface and connected to a SGE syringe (North Melbourne, Australia) by PEEK tubes (Fig. S1) [15]. After washing with water (all water washing steps were 5 min at 600 μL/h), TE microchips were deprotected with 1.2 mL hydrochloric acid (4 M, 200 μL/h) using a Cole Parmer 74,900 Series Single Syringe Pump. Deprotected microchips were washed with water, loaded with L-ascorbic acid (150 mg/mL in 66% methanol), and sealed for 30 min. The microchips loaded with L-ascorbic acid were labelled as thiol-ene-ascorbic acid (TEA) microchips and could be used for IgG glycopeptide enrichment. For protein digestion, TEA microchips were then washed with water, filled with 10 mg/mL trypsin, and incubated for 24 h at 4 °C. TE microchips immobilized with trypsin were labelled as TET microchips.

### Protein digestion

(1) Using TET microchips: after TET microchips were washed with water, 20 μL protein samples (all protein samples for digestion were in 10 mM NH_4_HCO_3_ in H_2_O, pH 8) were loaded at different flow rates (300 μL/h or 600 μL/h) and collected for MALDI-TOF–MS analysis. Between runs, the microchips were washed with 50 µL TA30 and then 50 µL H_2_O to get rid of protein/peptide residues. (2) Using *conventional batch method*: protein standard sample in 10 mM NH_4_HCO_3_ (pH 8.0) was incubated with trypsin at 37 °C for 17 h in thermomixer (Eppendorf ThermoMixer® C, Hamburg, Germany) at 1000 rpm. Trypsin/protein w/w ratio was 1:20. Digestion was terminated at 95 °C for 3 min. In the case of IgG extracted from human serum samples, IgG obtained from 5 μL human serum was incubated with 2 μg trypsin for digestion.

### Online TET microchip digestion and ESI–MS detection

To connect the microchip with ESI–MS (Bruker amaZon speed with ion trap) for online analysis, a capillary electrophoresis mass spectrometry (CE-MS) sprayer G1607 from Bruker was used. It is a triple tube sprayer with connection to a sample loading capillary, a sheath liquid delivery tube and a nebulizing gas introducing tube (Fig. S2). The outlet of the TET microchip was connected to a bare fused silica capillary (Polymicro Technologies, Phoenix, USA) with an inner/outer diameter of 75/365 μm via a tube adaptor. Two syringe pumps were used in the work, one was connected with the TET microchip for sample loading, and the other was connected to ESI–MS for introducing sheath liquid (80/20 acetonitrile/0.1% formic acid (v/v)). For online MS and MS/MS analysis, both sample loading flow rate and sheath liquid flow rate were set at 3 μL/min, capillary voltage was 3500 V, nebulizing gas was 1.2 bar, and nitrogen was used as dry gas at a flow rate of 8 L/min at 280 °C. Mass scan range was *m/z* 400–2000 for MS analysis and *m/z* 200–1800 for MS/MS analysis.

### IgG glycopeptide enrichment using TEA microchips

For method development, after washing with H_2_O, the TEA microchips were preconditioned with 50 μL loading solution (86% ACN/ 1% TFA v/v or 86% ACN/ 0.1% TFA v/v in H_2_O). After that, 20 μL IgG sample in loading solution (17.2 μL ACN, 0.2 μL TFA or 0.2 μL 10% TFA, 0.6 μL H_2_O, 2 μL of 1 mg/mL IgG digest) was loaded with a flow rate of 300 μL/h or 600 μL/h. The TEA microchips were then washed with 50 μL loading solution and eventually eluted with 50 μL H_2_O. The collected water fraction was marked as elution fraction and concentrated (Eppendorf concentrator 5301, Hamburg, Germany) to 3 μL for further analysis. When analyzing human serum IgG glycosylation, after washing and preconditioning (100 μL loading solution) the TEA microchip, 100 μL of sample solution containing 86% ACN, 1% TFA in H_2_O and 10 μL of IgG digest from 10 times diluted human serum IgG sample was loaded for IgG glycopeptide enrichment. After that, the microchip was washed with 100 μL loading solution and eluted with 100 μL H_2_O. The collected fraction was then concentrated to 10 μL for MALDI-TOF–MS analysis.

### IgG glycopeptide enrichment using HILIC tips

The 10μL HILIC tips (HyperSep Tip P-2 Type HILIC, Thermo Scientific, Rockwood, USA) were wetted with 5 times with 10 μL of H_2_O and conditioned 5 times with 10 μL of 86% ACN/1% TFA in H_2_O. After that, 10 μL of sample solution containing 2 μg IgG digest (2 μg IgG) and 86% ACN/1% TFA in H_2_O were loaded by aspirating and dispensing the sample solution for 20 times. After sample loading, HILIC tips were washed 5 times with 10 μL of 86% ACN/1% TFA in H_2_O. Finally, the glycopeptides were eluted with 10 μL of H_2_O and concentrated to 3 μL before MALDI-TOF–MS analysis.

### Biological samples and human serum IgG preparation

Human serum containing antibodies against SARS-COV-2 (EURM017) (CoV serum) was purchased from Sigma-Aldrich (Stockholm, Sweden). This commercial product is from donors 10 to 16 weeks after SARS-CoV-2 infection and tested negative to SARS-CoV-2. It has been pooled, delipidated, and sterilized according to the product instruction. Healthy human serum samples from two volunteers (laboratory staff, before the COVID-19 pandemic) were collected in connection to blood donation at a blood donation center and stored anonymously at  − 80 °C. The two non-identical healthy human serum samples were mixed and used for analysis. Human plasma IgG preparation procedures are referring to previously reported methods [13]. Briefly, human serum (5 μL) diluted with 20 mM Tris–HCl buffer (45 μL, pH 8.0) was loaded onto the Tris–HCl buffer preconditioned DEAE Affi-Gel® Blue Gel and mixed for 30 min in thermomixer at 1000 rpm. The supernatant containing IgG was collected as human serum IgG after centrifugation. Human serum IgG was then denatured at 95 °C for 10 min at 1000 rpm, reduced by DTT (10 mM) at 60 °C for 30 min, and alkylated by IAA (25 mM) in dark at 37 °C for 30 min in thermomixer. After reduction and alkylation, the sample solution was desalted using Pierce® C18 Tips. The desalted IgG sample was dried and reconstituted in H_2_O for trypsin digestion. The final sample volume was kept the same as the volume before denaturation.

### MALDI-TOF–MS and MALDI-TOF–MS/MS

0.5 µL sample obtained from digestion or enrichment, following the concentration step, was applied on a MALDI ground steel plate. 0.5 µL matrix was applied on the dried sample droplets. For each sample, 4 spots were prepared. After crystallization, sample spots were analyzed on an UltrafleXtreme MALDI TOF/TOF mass spectrometer (Bruker Daltonics, Bremen, Germany), which was equipped with a Smartbeam™-II laser (355 nm, UV). For peptide/glycopeptide analysis, 20 mg/mL 2,5-dihydroxybenzoic acid (DHB) in 30 ACN/70 0.1% TFA v/v (TA 30) was used as matrix, and samples were analyzed in positive reflectron MS mode, with laser intensity set to 70% and 4000 shots summed. Saturated α-cyano-4-hydroxycinnamic acid (HCCA) in TA 30 was used as matrix in MS/MS mode for fragmentation. In MS/MS mode, the laser intensity was set to 70% of the total intensity for parent ion and 90% for daughter ions. A single MS/MS spectrum was summed with 2000 shots for parent ion and 8000 shots for daughter ions.

### Peptide sequence determination

Software tools (Bruker Biotools) were used for peptide sequence determination. The MALDI-TOF–MS/MS spectra including fragmentation patterns were selected and opened with Biotools, by which the MS/MS mass lists were submitted to Mascot for search in the SwissProt database for MS/MS ions. Before the performance of a search, the following settings need to be selected or filled in: (1) the species: human; (2) missed cleavages: 0, 1, or 2 according to the peptide; (3) modifications: no amino acid modification; (4) MS tolerance: 50 ppm; (5) MSMS tolerance: 0.5 Da. After filling in all the information, the mass list can be searched against the database giving suggested matches with protein sequence information.

## Results and discussion

### Characterization of TE microchip using imaging

**Fig. 1:**
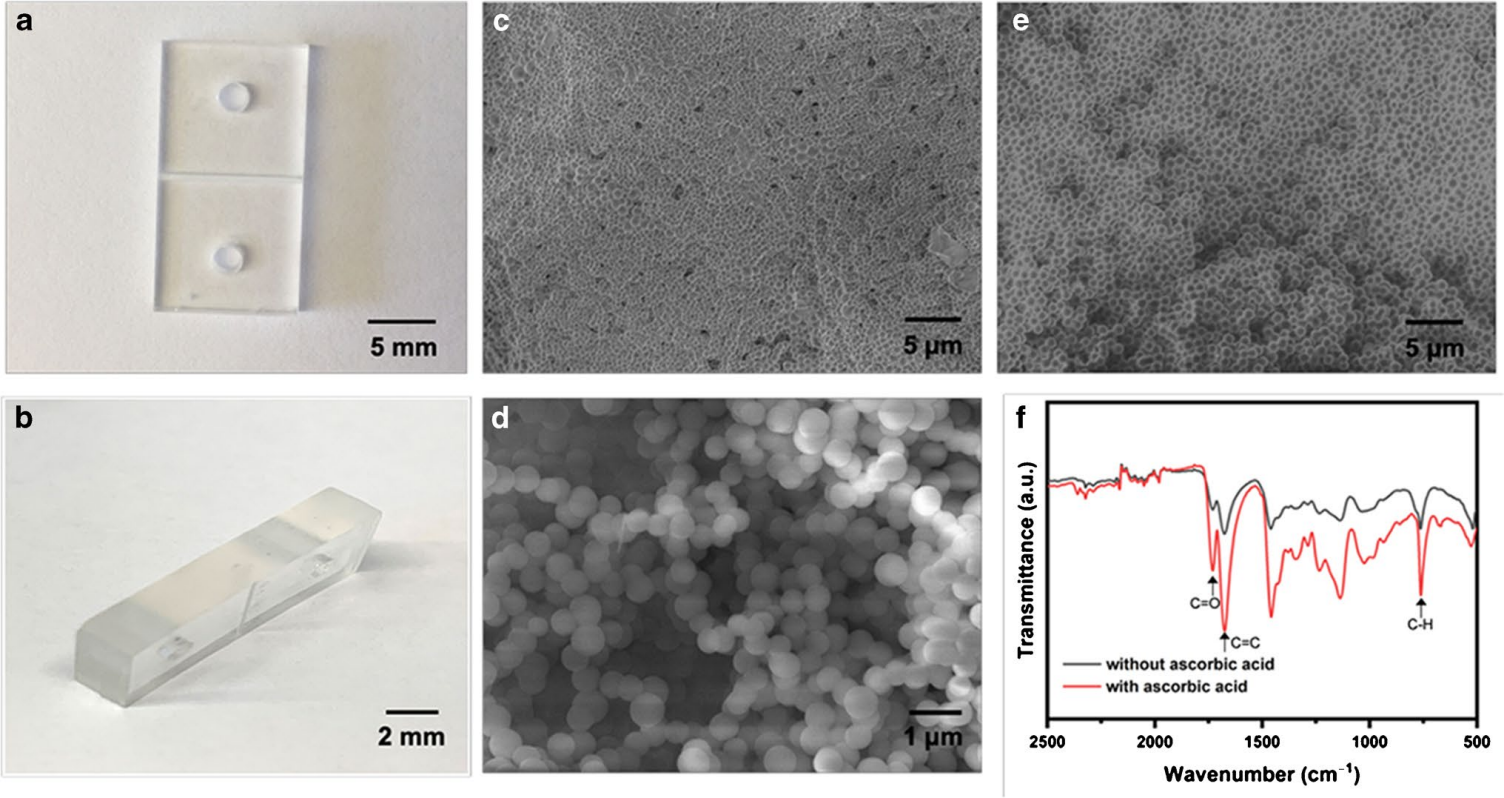
Images of a TE microchip. Optical images: **a** top view and **b** side view. SEM images of the monoliths with/without linking of ascorbic acid from top view: **c**–**d** without ascorbic acid, **c** 2.0 kv, 2500 × , **d** 15.0 kv, 11,000 × . **e** linked with ascorbic acid, 5.0 kv, 2500 × , **f** FTIR spectra of thiol-ene microchips with/without linking of ascorbic acid

The photographic images of a TE microchip are shown in Fig. 1a, b. A simple straight microchannel extending all the way to the edges with a “monolith” structure inside and dimensions of 1 cm long × 400 μm wide × 200 μm deep was fabricated. The monolith had a porous structure with “pores” in the µm size range (Fig. 1c, d), giving a density of 1.218 mg/μL, packing density of 60% (void volume of 40%), and a surface area of 21 cm^2^/mg, according to previously published work [15, 48]. It can be observed from Fig. 1d that the monolith structure consisted of interlinked beads with size of less than 1 μm in diameter. Figure 1c, d also shows the porous structure of the monolith increasing the surface-to-volume area, promoting the surface modification and enzyme immobilization inside of the monolith. After surface modification with ascorbic acid, the porous structure of the monolith was preserved and could be observed in Fig. 1e. The FTIR spectra in Fig. 1f show increased C = O stretching (1750–1735 nm), C = C stretching (1675–1665 nm), and C-H bending (750 ± 20 nm) for the microchip after linking with ascorbic acid, indicating the successful linking of ascorbic acid to the monolith.

### TET microchip trypsin digestion of rhOPN with flow rate optimization

Before loading protein samples, a blank run applying 10 mM NH_4_HCO_3_ was carried out to check if there was any material loss from the monolith. The MALDI-TOF–MS spectrum for a blank run in Fig. S3a showed neglectable noise from the monolith. A preliminary test with a TET microchip was carried out on a 500 µg/mL rhOPN sample solution at a flow rate of 600 µL/h for trypsin digestion. The flow rate of 600 µL/h, corresponding to a residence time of approximately 5 s, was preliminarily selected based on previous work where a flow rate of 780 µL/h was used [15]. Although no intact rhOPN protein could be detected after TET microchip digestion (Fig. S3b), the three most abundant peptide peaks (Fig. S3c) after digestion contain missed cleavages (MC), indicating insufficient digestion, likely due to the high concentration of the sample and the high flow rate. To obtain higher digestion efficiency, a sample concentration lower than 500 µg/mL is suggested, and a slightly lower flow rate is needed.

**Fig. 2:**
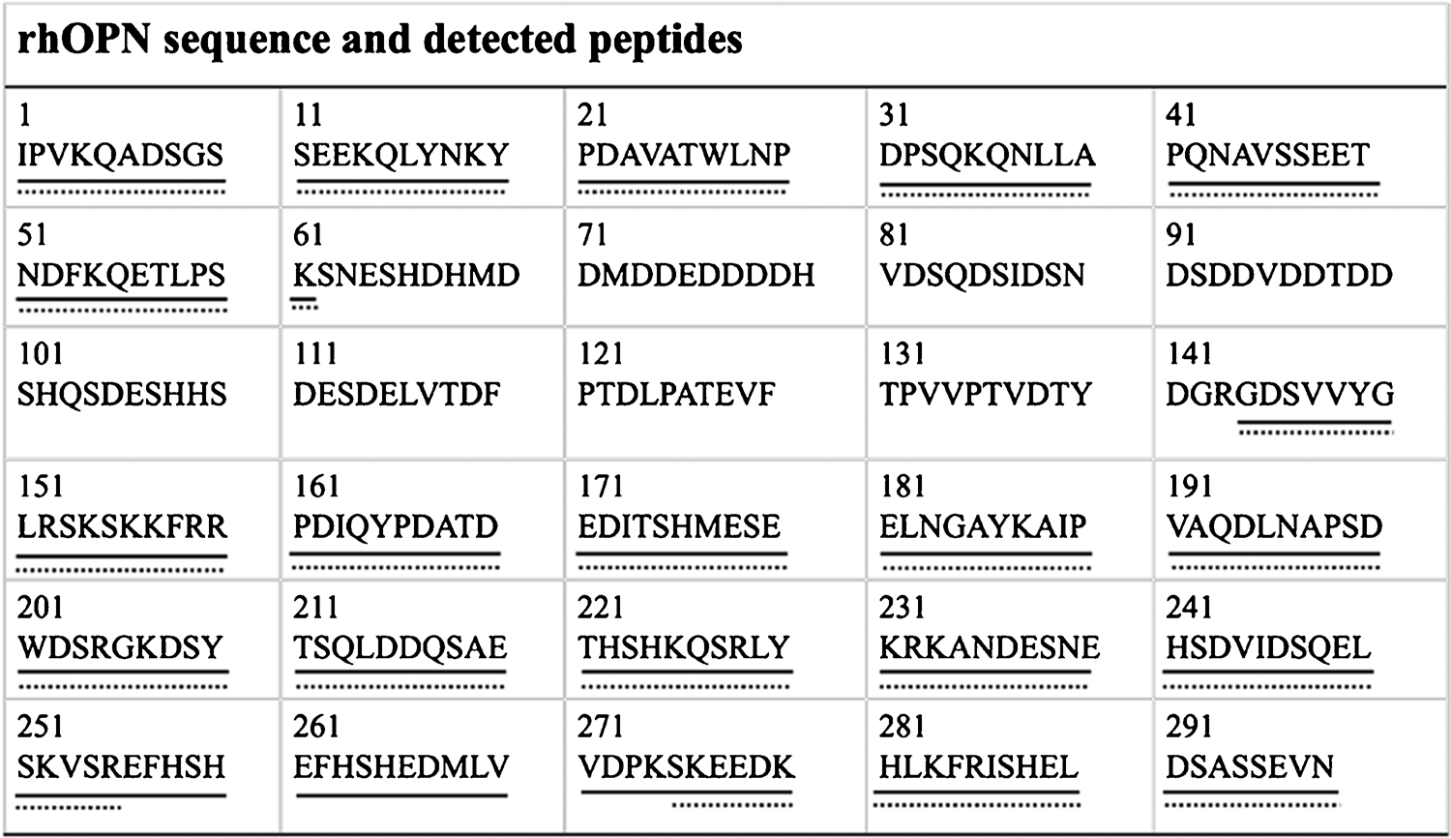
Sequence coverage of 100 µg/mL rhOPN digests using TET microchip at flow rates of 300 and 600 µL/h and detected with MALDI-TOF–MS. Detected peptides are underlined (solid line: 300 µL/h, sequence coverage 72.5%, dashed line: 600 µL/h, sequence coverage 66.1%)

A flow rate of 300 µL/h was investigated and compared with a flow rate of 600 µL/h, on 100 µg/mL rhOPN samples. There were in total 21 and 18 OPN peptides detected after TET microchip digestion at flow rates of 300 µL/h and 600 µL/h (Fig. S4), giving sequence coverages of 72.5% and 66.1%, respectively (Fig. 2). At a flow rate of 300 µL/h, the main peak in the spectrum was *m/z* 965.5 (144–152, GDSVVYGLR, MC: 0), while it was *m/z* 880.5 (153–159, SKSKKFR, MC: 3) at a flow rate of 600 µL/h. According to these results, higher digestion efficiency could be obtained when the flow rate was lower, due to the increased residence time. The only sequence that could not be detected at a flow rate of 300 µL/h was the one at the position of 62–143 with *m/z* 9179.6 (Fig. 2), having only one arginine (R) at the end of the chain, which means that this long peptide chain could not be further digested by trypsin. The rest of the rhOPN protein chain could be digested using the TET microchip at a flow rate of 300 µL/h (corresponding to a residence time of 5 s), however. Therefore, there was no further demand for even lower flow rate.

### TET microchip and conventional trypsin digestion of rhOPN

**Fig. 3:**
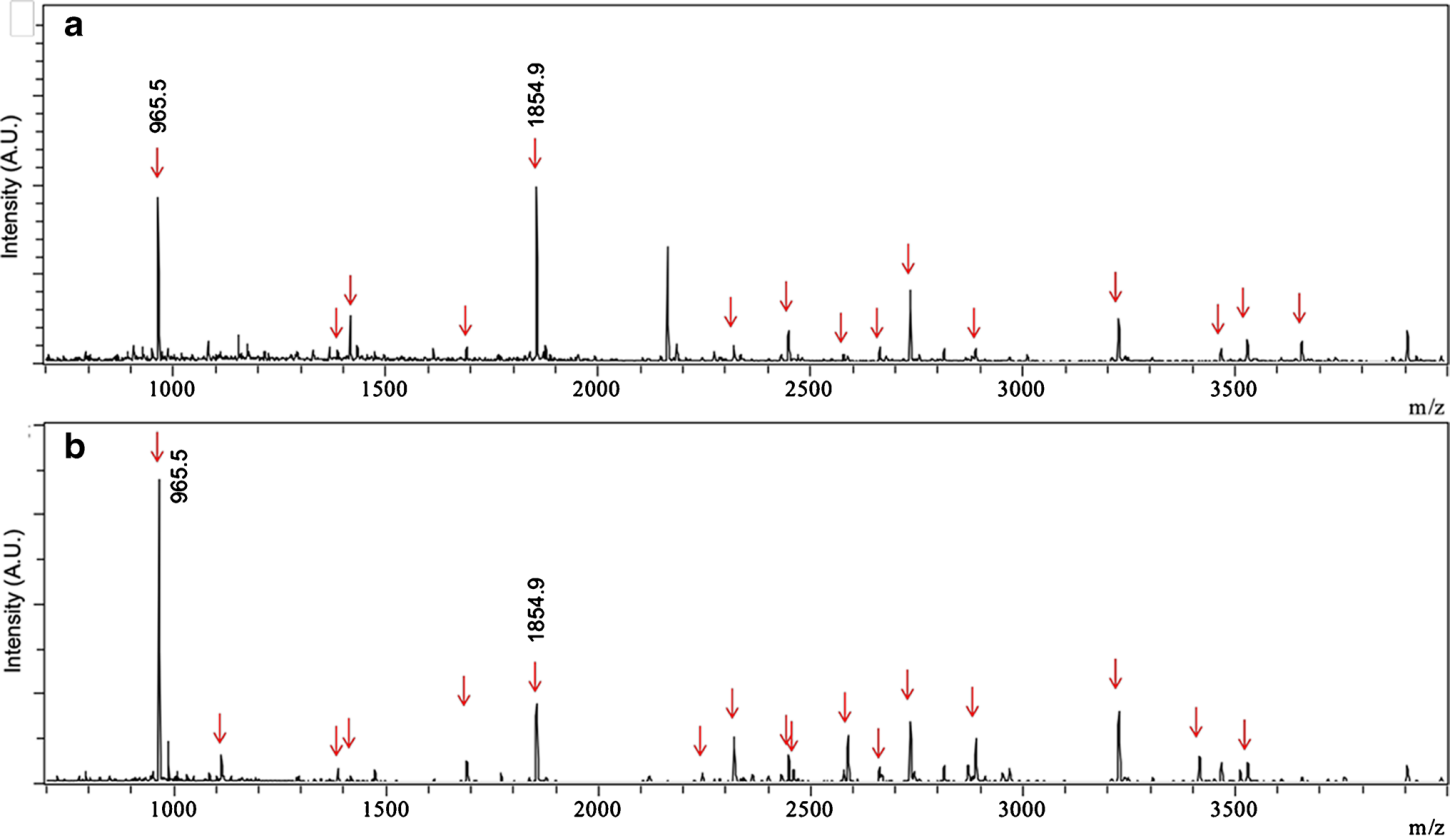
MALDI-TOF–MS spectra of 50 µg/mL rhOPN digests using **a** TET microchip digestion and **b** conv. TD (rhOPN/trypsin 20/1 w/w, detected OPN peptides are marked with red arrows)

The digestion efficiency of the TET microchip was compared with conv. TD on 50 µg/ml rhOPN samples. A total of 15 peptides could be detected after trypsin digestion using the TET microchip with a sequence coverage of 68.4%, while 17 peptides were detected with a sequence coverage of 64.7% using conv. TD (Fig. 3, Table S1). Thirteen peptides were detected with both digestion methods, and both methods showed the same main peaks of *m/z* 965.5 and 1854.9. TET microchips can be successfully used for rhOPN digestion, showing similar results as a 17 h conv. TD, but with residence times of only 10 s at a flow rate of 300 µL/h, corresponding to a roughly 6000-fold reduction in digestion time. One reason for the high digestion efficiency using TET microchip is probably the higher enzyme to protein ratio, which is possible to reach with immobilized enzymes. In the conventional digestion method, the mass ratio of trypsin/protein was 1/20. In the TET microchip, on the other hand, a 6.6 μg enzyme loading was estimated in the microchannel with 0.66 μL void volume, according to previous published data [15]. In the present work, the total volume of the monolith was 800 nL, and the loading amount of trypsin was calculated to be 3.2 μg (10 mg/mL), which was much higher than the concentration of loaded OPN proteins, resulting in a much higher ratio of trypsin/protein compared to the conventional digestion method.

### TET microchip repeatability within a single chip at different flow rates

**Fig. 4:**
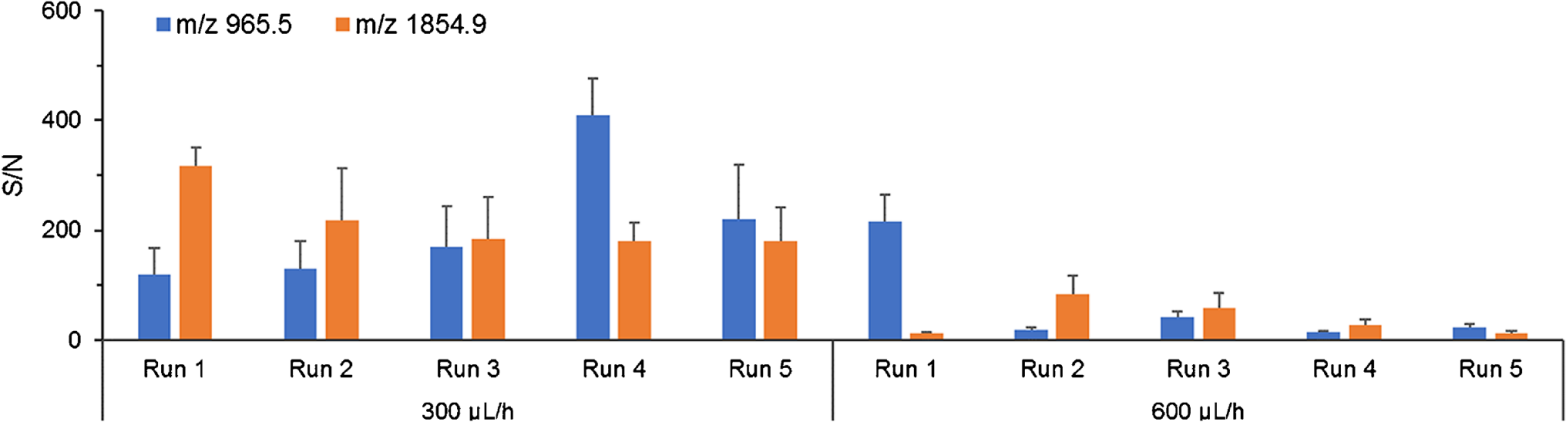
Average S/N values of OPN fingerprint peptides from consecutive runs using TET microchips with different flow rates. rhOPN concentration: 5 µg/mL, *n* = 8

In order to further investigate the digestion efficiency and within-chip repeatability, five consecutive runs on 5 µg/mL rhOPN samples were carried out within a single microchip at a certain flow rate (300 µL/h or 600 µL/h). Between runs, the microchips were washed first with TA 30 and then water to remove potential resides in the monolith. Collected water wash fractions showed trypsin autolysis peaks but no osteopontin peptides residues (Fig. S5), indicating sufficient washing between runs. At a flow rate of 300 µL/h, good repeatability could be obtained between the five consecutive runs, with the same main peaks (*m/z* 965.5 and 1854.9) (Fig. S5), similar numbers of detected peptides and similar sequence coverages (Table 1). The peptides with *m/z* 965.5 and 1854.9 (fingerprint peptides) have been used for the identification of OPN using MS/MS in previous works [49–51]. The sequences of these two peptides were further confirmed with MALDI-TOF–MS/MS spectra and Mascot database (Fig. S6). However, at a flow rate of 600 µL/h, the peak intensities of *m/z* 965.5 and 1854.9 became very low after three runs, and in some runs, the main peak was *m/z* 2888.4 with 1 MC (Fig. S7). Detected peptides in all runs are summarized in Table 1. It is obvious that both the number of detected peptides and the total sequence coverages were decreased after three runs at a flow rate of 600 µL/h. The average S/N values of the two fingerprint peptides from all runs are shown in Fig. 4; higher S/N values of the two peptides and better repeatability could be obtained when flow rate was 300 µL/h, instead of 600 µL/h.

**Table 1:** Total numbers and sequence coverages of detected OPN (5 µg/mL) peptides after trypsin digestion using TET microchips with flow rates of 300 µL/h and 600 µL/h

	Detected peptides (300 µl/h)					Detected peptides (600 µl/h)				
	Run 1	Run 2	Run 3	Run 4	Run 5	Run 1	Run 2	Run 3	Run 4	Run 5
Total number	17	18	18	17	18	14	11	12	11	8
Sequence coverage (%)	58.3	60.5	61.1	58.0	57.0	59.2	59.2	50.6	44.6	40.8

### TET microchip trypsin digestion of rhOPN at low concentrations

It is crucial to be able to detect samples with low concentrations when analyzing biological samples, due to the low abundance of many analytes, such as OPN, in the sample matrix. The concentrations of OPN have been reported to be lower than 100 ng/mL in serum/plasma of healthy people [52]. Elevated levels, up to more than 1 µg/mL, have been reported in many diseases, though [34–36, 38]. Here, three 100 ng/mL rhOPN samples were applied on three different chips. Two to 4 OPN peptides could be detected after TET microchip digestion, and the peptides with *m/z* 965.5 and 1854.9 could be detected in all runs (Fig. S8). The detection limits determined after rhOPN digest using a TET microchip are similar to what has been reported (100 ng/mL) in previous work using conventional TD and MALDI-TOF–MS detection [51]. According to the results obtained, it is suggested that the TET microchip could provide similar digestion performance for rhOPN at a low concentration, compared to the conventional digestion method.

### TET microchip trypsin digestion of other proteins

As described above, TET microchips were successfully assessed for the digestion of rhOPN, with similar performance as the conventional TD method. To further investigate the application of TET microchips for TD on other proteins, bovine serum albumin (BSA, ∼ 67 KDa) and IgG samples (∼ 152 KDa) were applied on TET microchips, respectively. Most of the peaks detected in conventionally digested BSA samples (100 µg/mL) can be detected in TET microchip digested samples (Fig. S9), and around 28% of the total sequence could be detected using the TET microchips as well as when using conventional TD. When analyzing IgG samples (100 µg/mL), IgG could be digested by TET microchip, and some glycopeptides could be directly detected on MALDI-TOF–MS in some runs, while in other runs, intact IgG could still be detected (Fig. S10). The repeatability of IgG digestion using TET microchip is thus not as good as for rhOPN, probably due to the large size and the complexity of the IgG structure.

### Online TET microchip trypsin digestion and ESI–MS detection

The TET microchip was also evaluated for online protein digestion. TET microchips were connected to ESI–MS for introducing the digests directly into the MS ion source for online digestion and detection. The ESI–MS spectra for online BSA and rhOPN digests are shown in Fig. S11(a and c), with MS/MS analysis in Fig. S11(b and d), respectively. Compared to MALDI-TOF–MS spectra, less digested peptides were detected with ESI–MS for both protein digests, probably due to the background noise, the presence of salt in samples, the charge states of the peptides, and the performance of the instrument. The peptides are singly charged in MALDI-TOF–MS and detected at a higher mass range to minimize the interference from low mass components, e.g., from the matrix, while the peptides are multiply charged in ESI–MS and detected at a lower mass range, which could affect the detection of some peptides. A way to avoid the influence of the noise peaks on the identification is to analyze the daughter ions of selected parent ions could help. For instance, the main peak ions (*m/z* 820 with charge of 2 + for BSA and *m/z* 928 with charge of 2 + for rhOPN) were selected as parent ions for further MS/MS fragmentation. The MS/MS spectra of the parent ions showed clear fragmentation patterns, and the identification results are shown in Fig. S11 (b and d). Both proteins could be successfully online digested and detected online, providing rapid analysis within minutes.

### TEA microchip IgG glycopeptide enrichment

**Fig. 5:**
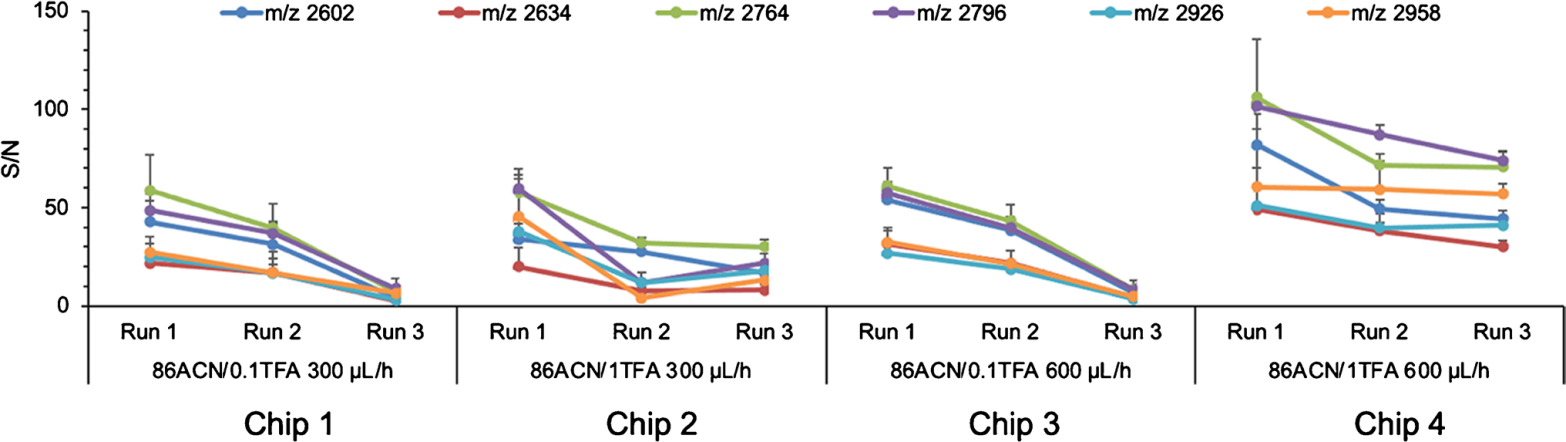
The average S/N values of the 6 most abundant glycopeptides in all runs after glycopeptide enrichment from 2 µg IgG digest using TEA microchips. Five spots on the MALDI plate were analyzed for each sample

When using a TE chip without ascorbic acid modification, no glycopeptide enrichment was observed. After adding an ascorbic acid linker on the surface of the thiol-ene monolith, the acidic functionality of ascorbic acid made the surface of the monolith hydrophilic, offering the possibility to perform glycopeptide enrichment according to the mechanism of hydrophilic interaction chromatography. In studies of glycopeptide enrichment, loading solutions containing 83 ~ 86% ACN with 0.1 ~ 1% TFA in H_2_O have commonly been used, and H_2_O has been used to elute the glycopeptides from hydrophilic materials [13, 46, 53, 54]. Here, 86% ACN/0.1%TFA (v/v), and 86% ACN/1%TFA (v/v) in H_2_O were evaluated as loading solutions. Two microgram IgG digest in loading solution was applied on TEA microchips and eluted with H_2_O. Flow rates of 300 µL/h and 600 µL/h were compared. In total, four microchips were used, and three or four consecutive runs were performed for the same sample in each microchip. MALDI-TOF–MS spectra of elution fractions from all runs are shown in Figs. S12 and S13. Almost all non-glycopeptides could, in all runs, be separated from the glycopeptides by enrichment using the TEA microchips. The determination of IgG glycopeptides in present work was carried out by referring to our previous work where the same batch of samples were analyzed [13]. The average S/N values of the 6 most abundant glycopeptides in all runs are compared in Fig. 5. It can be seen that the highest S/N values of the selected glycopeptides were obtained using Chip 4 (loading solution: 86% ACN/1%TFA (v/v) in H_2_O, flow rate: 600 µL/h), compared to other chips. A fourth run was also carried out on Chip 4, but some non-glycopeptides started to appear in the spectrum (Fig. S13g) and the S/N values of the 6 most abundant glycopeptides decreased with the number of runs (Fig. S13h). Therefore, a loading solution of 86% ACN/1%TFA (v/v) at a flow rate of 600 µL/h and maximum of three consecutive runs in the same chip are recommended for IgG glycopeptide enrichment using TEA microchips. The limit of detection using TEA chips has also been assessed by loading IgG digests with lower concentrations (Fig. S14). Twelve glycopeptides could be detected when 40 µg/ml IgG digest was loaded, corresponding to 100 fmol/spot. Four glycopeptides could still be detected when the loading concentration of IgG digest was 10 µg/ml, corresponding to only 25 fmol/spot, showing a good limit of detection for this enrichment method.

**Fig. 6:**
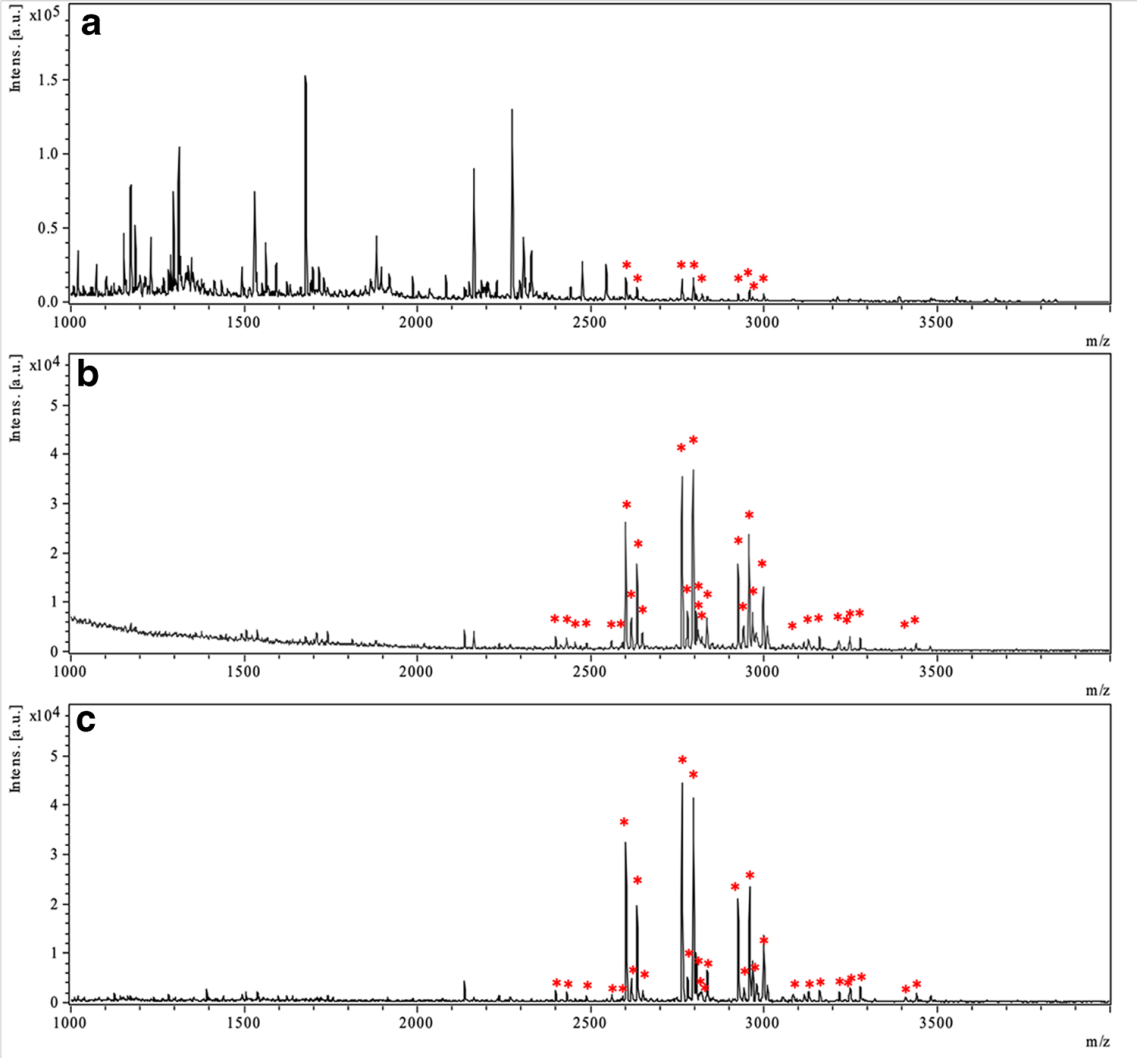
MALDI-TOF–MS of **a** IgG digest without enrichment. **b** Glycopeptides from 2 μg IgG digest enriched using TEA microchips. **c** Glycopeptides from 2 μg IgG digest enriched using HILIC tips. Glycopeptides are marked with stars

Glycopeptide enrichment selectivity obtained when using TEA microchips was compared with a commercial HILIC product. MALDI-TOF–MS spectra of IgG digest without glycopeptide enrichment and enriched glycopeptides using these two different materials are shown in Fig. 6. All enriched IgG glycopeptides detected using the different methods are listed in Table S2 with glycan composition and amino acid sequence information. The glycan compositions and peptide sequence information were obtained by referring to previous studies [13, 54]. Most of the non-glycopeptides can be separated from the glycopeptides when using both materials, giving similar selectivity. After enrichment, 31 glycopeptides with 14 glycan compositions, including 15 glycopeptides from IgG1 and 16 from IgG2, could be enriched by TEA microchips and detected using MALDI-TOF–MS. Thirty glycopeptides with 14 glycan compositions, including 15 glycopeptides from IgG1 and 15 from IgG2, were enriched and detected when using HILIC tips. Both of the materials could enrich glycopeptides with a range of *m/z* values from 2300 up to 3500, including glycopeptides with a large variety in glycan structure. Besides glycopeptides with 0 missed cleavages in the peptide chain, glycopeptides with 1 missed cleavage could also be enriched and detected using both materials. It also could be observed from Fig. 6 and Table S2 that all of the 6 most abundant glycopeptides are fucosylated and most of the detected glycopeptides are galactosylated. TEA microchip exhibited a high similarity in selectivity in glycopeptide enrichment compared to the commercial HILIC product, while providing possibility of online analysis.

### TEA microchip IgG glycopeptide enrichment in human serum samples

**Fig. 7:**
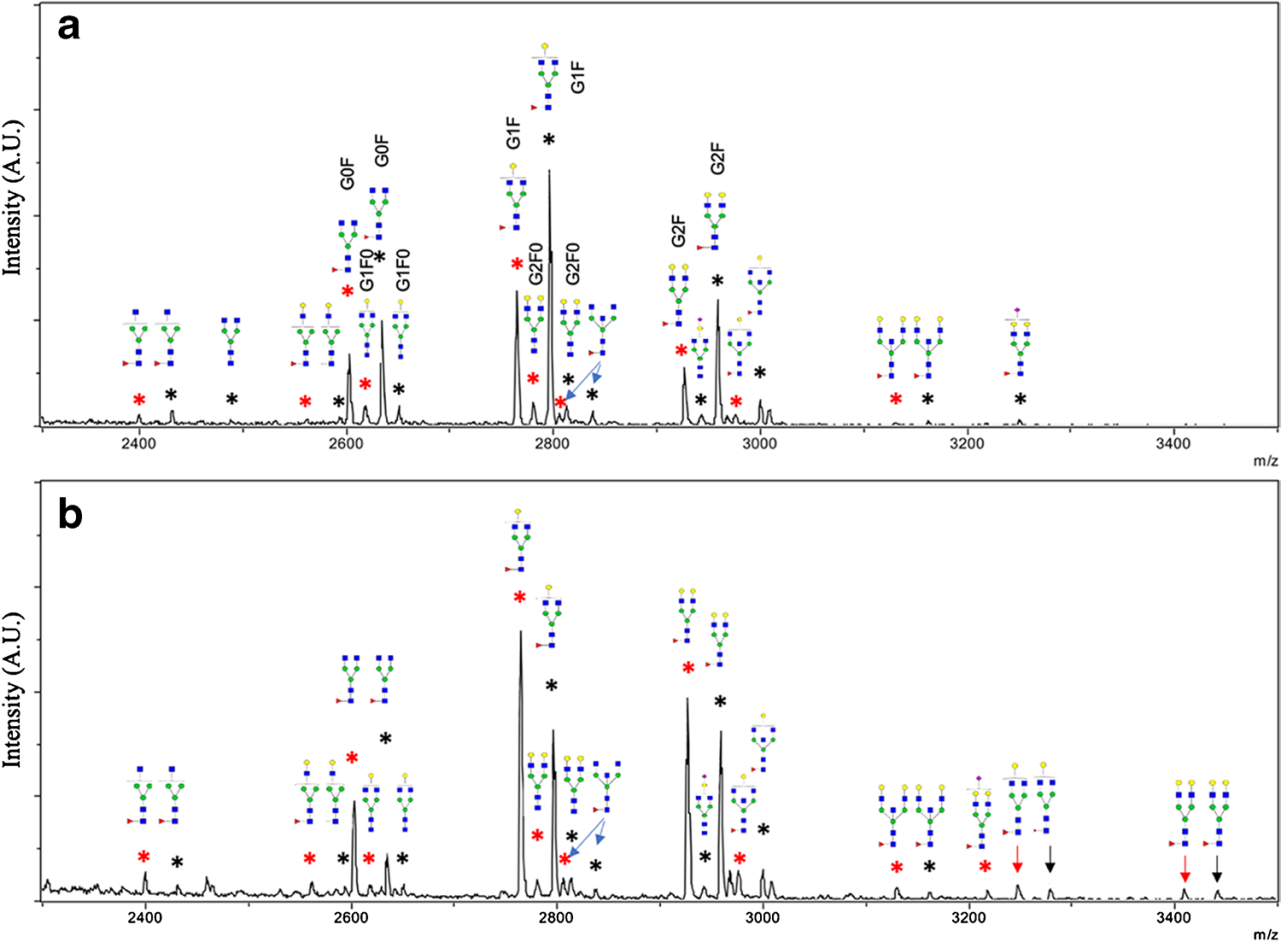
MALDI-TOF–MS spectra of enriched glycopeptides from human serum IgG samples, **a** CoV serum, **b** healthy serum. Red star, peptide sequence EEQFNSTFR (IgG2); black star, peptide sequence EEQFNSTYR (IgG1); red arrow in **b,** peptide sequence TKPREEQFNSTFR (IgG2); black arrow in **b**, peptide sequence TKPREEQFNSTYR (IgG1). Loading solution: 86% ACN/1%TFA (v/v), flow rate: 600 µL/h. In glycan structure, blue square, N-acetylglucosamine; red triangle, fucose; green circle, manose; yellow circle, galactose; purple diamond, sialic acid

To further investigate the glycopeptide enrichment performance, the microchips were used on real samples representing more complex matrices, containing a variety of components that potentially could disturb the enrichment process. IgG samples from healthy and CoV human sera were analyzed respectively. For each serum IgG sample, a new TEA microchip was used for glycopeptide enrichment after the IgG sample was pretreated and digested. The detection of glycopeptides is obscured in IgG digests from both serum samples; maximum five glycopeptides could be detected with very low intensities (Fig. S15). However, using TEA microchip glycopeptide enrichment, most of the non-glycopeptides were not retained in the microchip and could be removed during loading and washing. This resulted in enriched IgG glycopeptides in the elution fraction with substantially improved detection (Fig. S15). The MALDI-TOF–MS spectra of enriched and detected glycopeptides from CoV serum IgG digest and healthy serum IgG digest are shown in Fig. 7. Most of the glycoforms detected in healthy serum samples can be detected in CoV serum samples; nevertheless, some differences in fucosylation and galactosylation of those two samples can be observed. There are four glycopeptides with one missed cleavage (two from IgG1 and two from IgG2) that could be detected in healthy serum sample but not in CoV serum sample. Those two glycoforms are both fucosylated and galactosylated. There is one glycoform (glycopeptide *m/z* 2488.0) that could be detected in CoV serum but not in healthy serum sample. This glycoform is non-fucosylated and non-galactosylated.

The changes in IgG overall glycosylation in CoV serum samples could be due to its association with inflammatory responses in COVID-19. Elevated levels of non-fucosylation and non-galactosylation in COVID-19 antibodies have been reported in previous studies [6, 7]. However, to conduct a full comparison study between healthy serum samples and CoV serum samples, a higher number of different samples would be required, which is outside the scope of this work. The results obtained in the present work clearly indicate the potential of the use of TEA microchip in glycosylation studies of complex biological samples.

## Conclusion

TE microchips immobilized with trypsin were successfully utilized for trypsin digestion of rhOPN, showing similar performance as conventional digestion methods, while the TET microchip could offer rapid digestion and online analysis within minutes. This online digestion setup together with MS/MS analysis could thus provide higher throughput for future studies of OPN-related diseases. TE microchips surface modified with an ascorbic acid linker were successfully used for the enrichment of IgG glycopeptides from IgG standard and human serum samples. IgG glycopeptides from healthy human serum and CoV human serum samples were successfully enriched using TEA microchips. Our experiments demonstrated new applications of functionalized high internal surface TE microchips in proteomics and thus expand the possibilities of using such microfluidic chips for a variety of purposes. The two functions demonstrated here individually could potentially be applied simultaneously in future work for online protein digestion and glycopeptide enrichment. This would further increase the time-to-result efficiency of the whole procedure, benefiting the analysis of biological samples.

## Supplementary Information

Below is the link to the electronic supplementary material.

Supplementary file1 (PDF 5106 KB)

## Declarations

### Use of human serum samples from healthy volunteers

As the serum samples were pooled anonymized samples from non-identical volunteers, they cannot be traced back to an individual person. Thus, any ethical permit is not required according to the scope (Sects. 3–5) of the Swedish Ethical Review Act.
