# Supplementary material for: Thiol-ene-based microfluidic chips for glycopeptide enrichment and online digestion of inflammation-related proteins osteopontin and immunoglobulin G
Source: Anal Bioanal Chem. 2023 Jan 6;415(6):1173–85. doi: 10.1007/s00216-022-04498-2 (PMC9817458; doi:10.1007/s00216-022-04498-2)
Supplement: Supplementary file 1 — Supplementary file1 (PDF 5106 KB) [file 216_2022_4498_MOESM1_ESM.pdf]

Supplementary Information

Thiol-ene based microfluidic chips for glycopeptide enrichment and online digestion of inflammation-related proteins osteopontin and immunoglobulin G†

Yuye Zhou,[a] Alexander Jönsson,[b] Drago Sticker,[c] Guojun Zhou,[d] Zishuo Yuan,[e] Jörg P. Kutter,[e] * Åsa Emmer [a] *

[a] KTH Royal Institute of Technology, School of Engineering Sciences in Chemistry, Biotechnology and Health, Department of Chemistry, Analytical Chemistry, 100 44 Stockholm, Sweden

[b] Technical University of Denmark, Department of Health Technology, 2800 Kongens Lyngby, Denmark

[c] Novo Nordisk A/S, Biophysics and Formulation, 2760 Måløv, Denmark

[d] Stockholm University, Department of Materials and Environmental Chemistry, 106 91 Stockholm, Sweden

[e] University of Copenhagen, Department of Pharmacy, 2100 Copenhagen, Denmark

† Supplementary Information

* Corresponding authors: aae@kth.se, jorg.kutter@sund.ku.dk

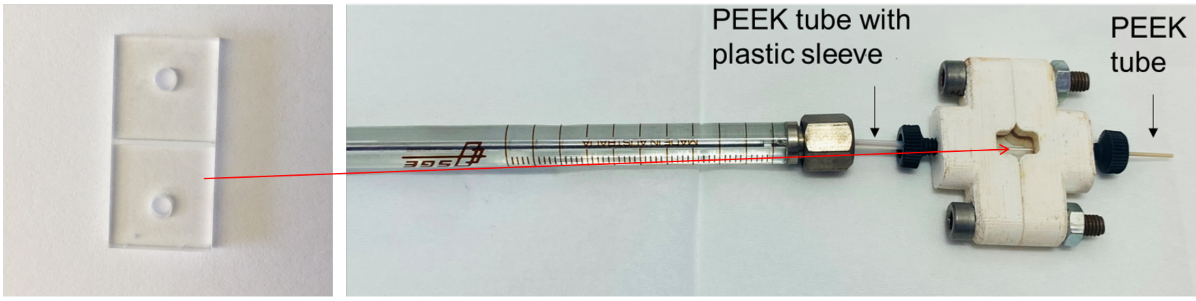

Figure S1. Images of a TE microchip (left) and microchip connection set up (right).

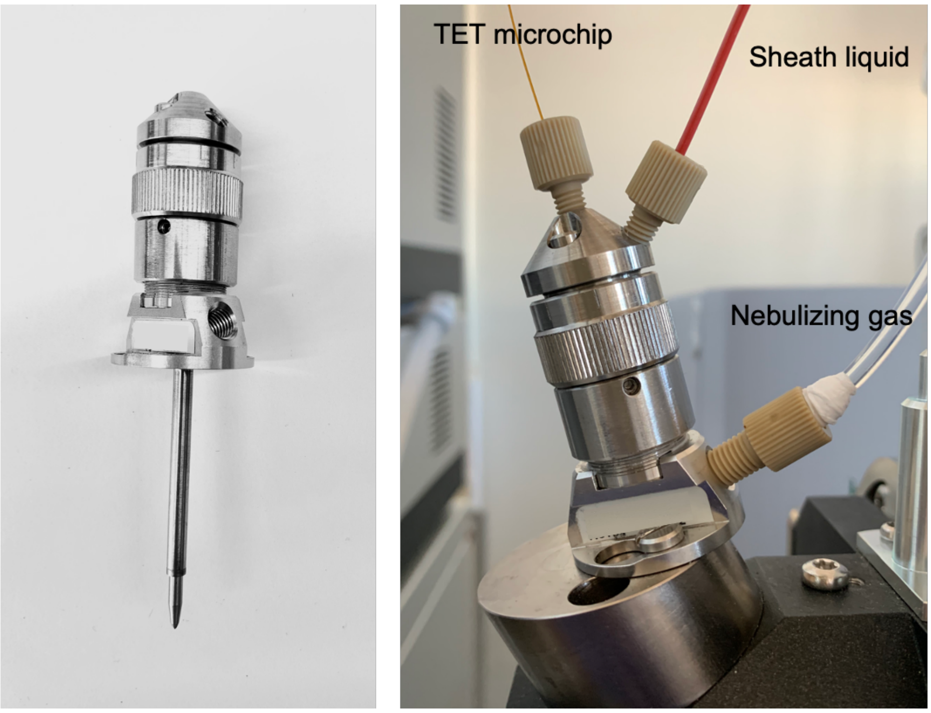

Figure S2. Images of G1607 sprayer and the connections of the sprayer to TET microchip, sheath liquid and nebulizing gas.

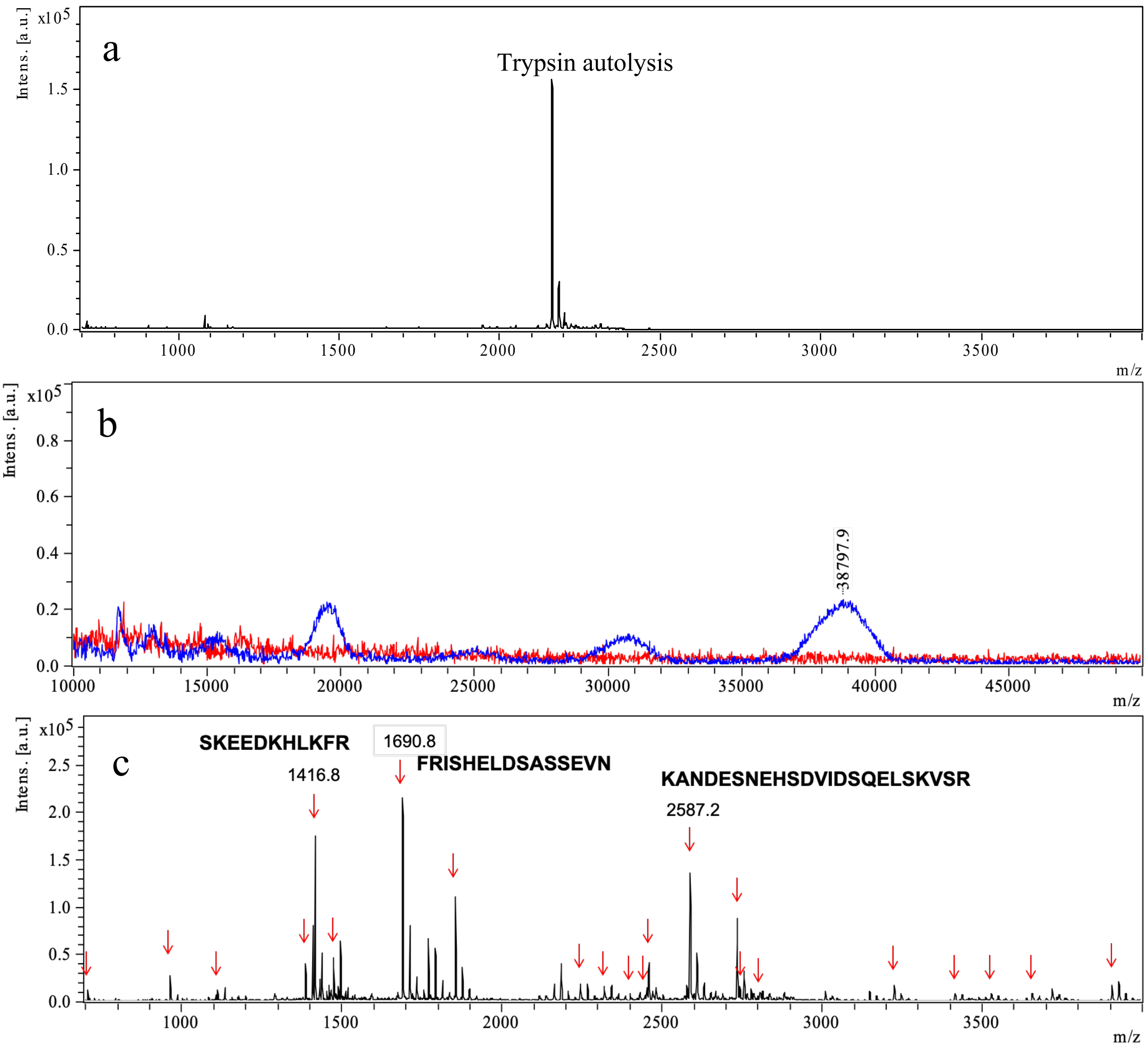

10 20 30 40 50 60 70 80
IPVKQADSGS SEEKQLYNKY PDAVATWLNP DPSQKQNLLA PQNAVSSEET NDFKQETLPS KSNESHDHMD DMDDEDDDDH
90 100 110 120 130 140 150 160
VDSQDSIDSN DSDDVDDTDD SHQSDESHHS DESDELVTDF PTDLPATEVF TPVVPTVDTY DGRGDSVVYG LRSKSKKFRR
170 180 190 200 210 220 230 240
PDIQYPDATD EDITSHMESE ELNGAYKAIP VAQDLNAPSD WDSRGKDSYE TSQLDDQSAE THSHKQSRLY KRKANDESNE
250 260 270 280 290
HSDVIDSQEL SKVSREFHSH EFHSHEDMLV VDPKSKEEDK HLKFRISHEL DSASSEVN

Figure S3. a) Blank MALDI-TOF-MS run (10 mM NH_4HCO_3). b and c) MALDI-TOF-MS spectra of 500 µg/ml rhOPN and detected OPN peptides after digestion. b): mass range *m/z* 10000 to 50000 , red: after digestion using TET chip, blue: before digestion. c): mass range *m/z* 700 to 4000 , rhOPN after digestion using TET microchip at a flow rate of 600 µL/h. Detected OPN peptides are marked with red arrows in b) and underlined in the sequence. rhOPN intact protein mass: 39 kDa.

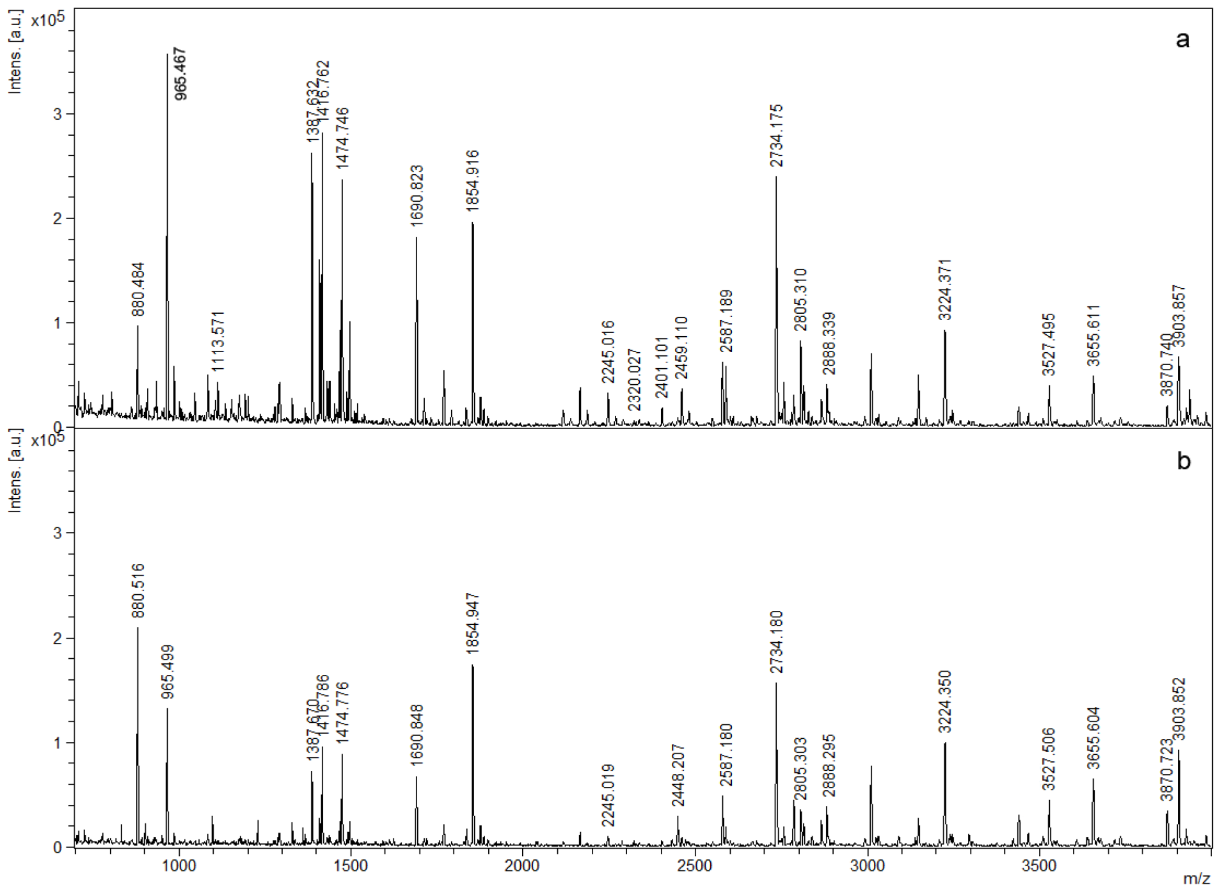

Figure S4. MALDI-TOF-MS spectra of 100 μg/mL rhOPN digests using TET chips with a flow rate of a) 300 μL/h and b) 600 μL/h. All peaks with labels are OPN digest peptides.

Table S1. Theoretical and detected OPN peptides after trypsin digestion of 50 µg/mL rhOPN samples using: a) TET chip, flow rate 300 µL/h, 10s, b) conventional TD, rhOPN/trypsin 20/1 (w/w), 17 h.

No.	m/z of OPN digests Theoretical	a	b	Sequence	Position	Missed cleavage
1	965.5	965.5	965.5	GDSVVYGLR	144-152	0
2	1113.6	/	1113.6	SKEEDKHLK	275-283	2
3	1387.6	1387.6	1387.7	ISHELDSASSEVN	286-298	0
4	1416.8	1416.8	1416.8	SKEEDKHLKFR	275-285	3
5	1690.8	1690.8	1690.8	FRISHELDSASSEVN	284-298	1
6	1854.9	1854.9	1854.9	AIPVAQDLNAPSDWDSR	188-204	0
7	2245.0	/	2245.0	KANDESNEHSDVIDSQELSK	233-252	1
8	2320.0	2320.0	2320.0	EFHSHEFHSHEDMLVVDPK	256-274	0
9	2448.2	2448.2	2448.2	QLYNKYPDAVATWLNPDPSQK	15-35	1
10	2459.1	/	2459.1	ANDESNEHSDVIDSQELSKVSR	234-255	1
11	2587.2	2587.2	2587.2	KANDESNEHSDVIDSQELSKVSR	233-255	2
12	2662.2	2662.2	2662.2	VSREFHSHEFHSHEDMLVVDPK	253-274	1
13	2734.2	2734.2	2734.2	GKDSYETSQLDDQSAETHSHKQSR	205-228	2
14	2888.4	2888.3	2888.3	QNLLAPQNAVSSEETNDFKQETLPSK	36-61	1
15	3224.5	3224.4	3224.4	RPDIQYPDATDEDITSHMESEELNGAYK	160-187	0
16	3414.6	/	3414.6	EFHSHEFHSHEDMLVVDPKSKEEDKHLK	256-283	3
17	3466.6	3466.6	/	QADSGSSEEKQLYNKYPDAVATWLNPDPSQK	5-35	2
18	3527.6	3527.5	3527.5	FRRPDIQYPDATDEDITSHMESEELNGAYK	158-187	1
19	3655.7	3655.6	/	KFRRPDIQYPDATDEDITSHMESEELNGAYK	157-187	2

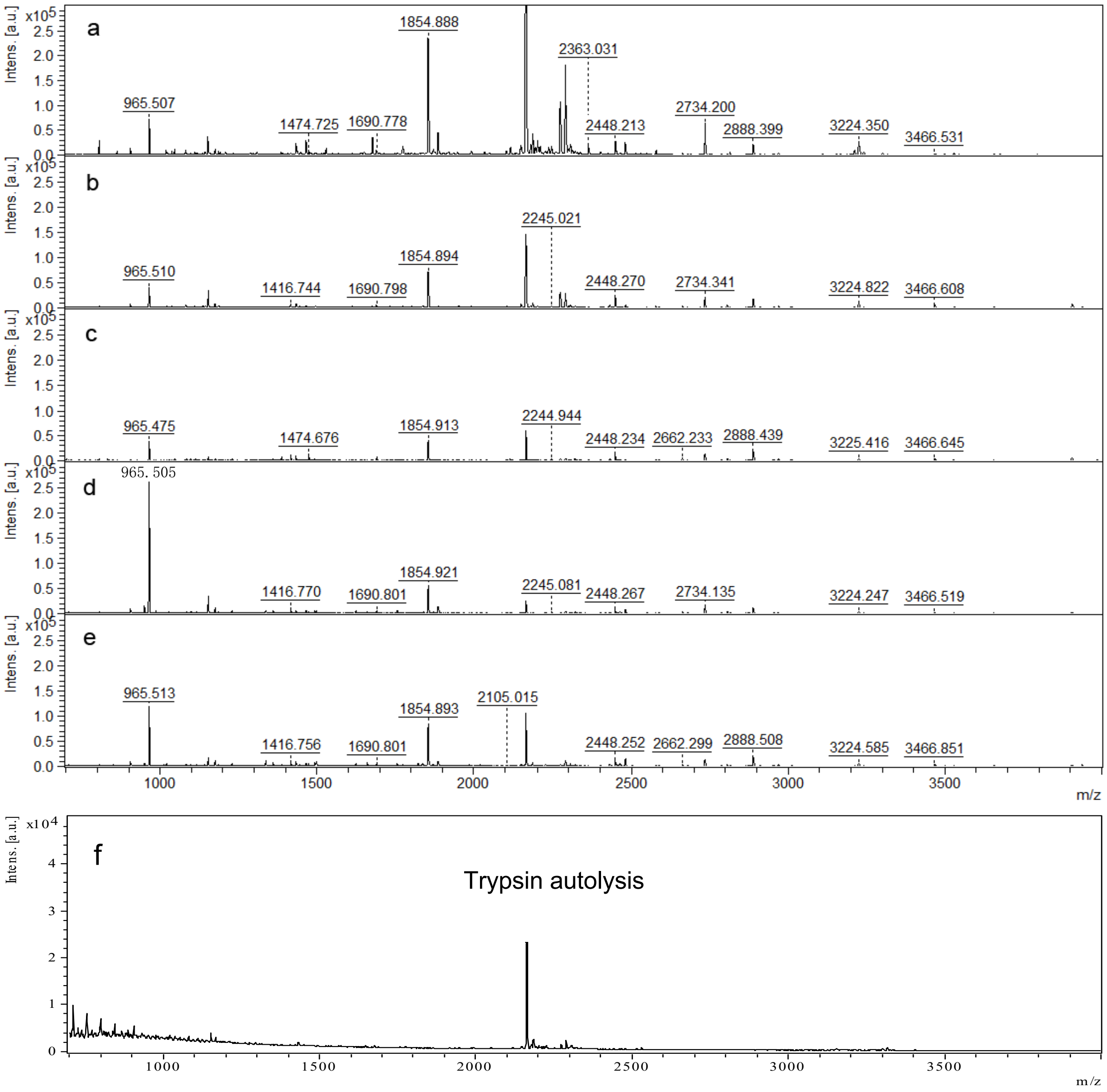

Figure S5. MALDI-TOF-MS spectra of 5 μg/mL rhOPN digests obtained from five consecutive runs using the same TET chip with flow rate 300 μL/h. a) run 1, b) run 2, c) run 3, d) run 4 and e) run 5. All peaks with labels are OPN digest peptides. f) MALDI-TOF-MS spectrum of H_2O wash fraction after “run 1”.

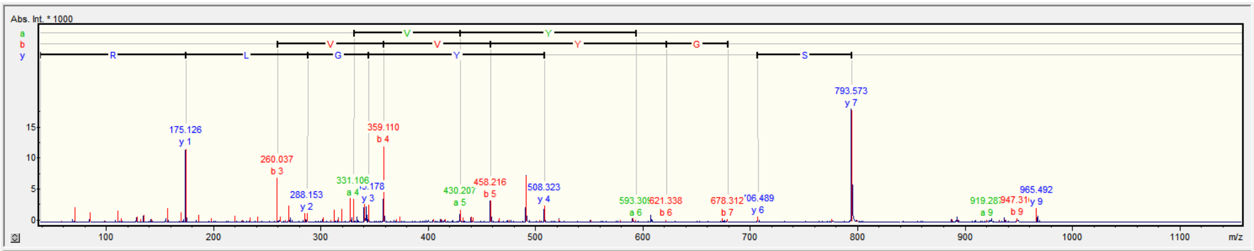

Protein View | Match Errors | MS/MS Fragments | MSMS Analysis

Sequence: GDSVVYGLR (Mods:)

Masses: MH+(mono): 965.505 MH+(avg): 966.071 MS/MS Tol: 0.500 Da Peaks: 69 Above Threshold: 69 Zoom to m/z for 2+ 1+ Zoom +/-: 10.0 Da

Monoisotopic Average Calculate: Masses Threshold: 0.000 Assigned: 33 Not Assigned: 36 1+, m/z: 0.000

	G	D	S	V	V	Y	G	L	R	Gly	Asp	Ser	Val	Val	Tyr	Gly	Leu	Arg
Ion	1	2	3	4	5	6	7	8	9	1	2	3	4	5	6	7	8	9
a	G	D	S	V	V	Y	G	L	R	30.034	145.061	232.093	331.161	430.230	593.293	650.314	763.398	919.500
b	G	D	S	V	V	Y	G	L	R	58.029	173.056	260.088	359.156	458.225	621.288	678.309	791.393	947.495
a-17	G	D	S	V	V	Y	G	L	R	13.007	128.034	215.066	314.135	413.203	576.266	633.288	746.372	902.473
a-18	G	D	S	V	V	Y	G	L	R	12.023	127.050	214.082	313.151	412.219	575.282	632.304	745.388	901.489
b-17	G	D	S	V	V	Y	G	L	R	41.002	156.029	243.061	342.130	441.198	604.261	661.283	774.367	930.468
b-18	G	D	S	V	V	Y	G	L	R	40.018	155.045	242.077	341.146	440.214	603.277	660.299	773.383	929.484
b+18	G	D	S	V	V	Y	G	L	R	76.039	191.066	278.098	377.167	476.235	639.298	696.320	809.404	965.505
y	G	D	S	V	V	Y	G	L	R	175.119	288.203	345.224	508.288	607.356	706.425	793.457	908.484	965.505
y-17	G	D	S	V	V	Y	G	L	R	158.116	271.200	328.222	491.285	590.353	689.422	776.454	891.481	948.502
i	G	D	S	V	V	Y	G	L	R	30.034	88.039	60.044	72.081	72.081	136.076	30.034	86.096	129.113
	9	8	7	6	5	4	3	2	1	Arg	Leu	Gly	Tyr	Val	Val	Ser	Asp	Gly

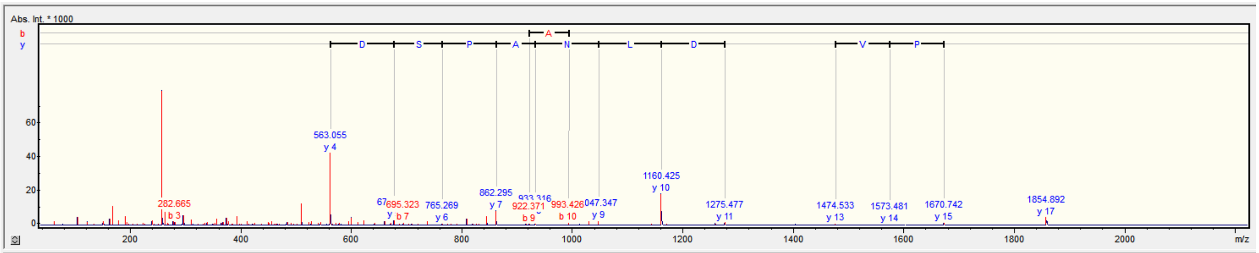

Protein View | Match Errors | MS/MS Fragments | MSMS Analysis

Sequence: AIPVAQDLNAPSDWDSR (Mods:)

Masses: MH+(mono): 1854.898 MH+(avg): 1855.981 MS/MS Tol: 0.500 Da Peaks: 134 Above Threshold: 134 Zoom to m/z for 2+ 1+ Zoom +/-: 10.0 Da

Monoisotopic Average Calculate: Masses Threshold: 0.000 Assigned: 30 Not Assigned: 104 1+, m/z: 0.000

	A	I	P	V	A	Q	D	L	N	A	P	S	D	W	D	S	R
Ion	1	2	3	4	5	6	7	8	9	1	1	1	1	1	1	1	1
a	A	I	P	V	A	Q	D	L	N	A	P	S	D	W	D	S	R
b	A	I	P	V	A	Q	D	L	N	A	P	S	D	W	D	S	R
a-17	A	I	P	V	A	Q	D	L	N	A	P	S	D	W	D	S	R
a-18	A	I	P	V	A	Q	D	L	N	A	P	S	D	W	D	S	R
b-17	A	I	P	V	A	Q	D	L	N	A	P	S	D	W	D	S	R
b-18	A	I	P	V	A	Q	D	L	N	A	P	S	D	W	D	S	R
b+18	A	I	P	V	A	Q	D	L	N	A	P	S	D	W	D	S	R
y	A	I	P	V	A	Q	D	L	N	A	P	S	D	W	D	S	R
y-17	A	I	P	V	A	Q	D	L	N	A	P	S	D	W	D	S	R
i	A	I	P	V	A	Q	D	L	N	A	P	S	D	W	D	S	R
	1	1	1	1	1	1	1	1	9	8	7	6	5	4	3	2	1

	Ala	Ile	Pro	Val	Ala	Gln	Asp	Leu	Asn	Ala	Pro	Ser	Asp	Trp	Asp	Ser	Arg
Ion	1	2	3	4	5	6	7	8	9	10	11	12	13	14	15	16	17
a	44.049	157.134	254.186	353.255	424.292	552.350	667.377	780.461	894.504	965.541	1062.594	1149.626	1264.653	1450.733	1565.759	1652.791	1808.893
b	72.044	185.128	282.181	381.250	452.287	580.345	695.372	808.456	922.499	993.536	1090.589	1177.621	1292.648	1478.727	1593.754	1680.786	1836.888
a-17	27.023	140.107	237.160	336.228	407.265	535.324	650.351	763.435	877.478	948.515	1045.568	1132.600	1247.627	1433.706	1548.733	1635.765	1791.866
a-18	26.039	139.123	236.176	335.244	406.281	534.340	649.367	762.451	876.494	947.531	1044.584	1131.616	1246.643	1432.722	1547.749	1634.781	1790.882
b-17	55.018	168.102	265.155	364.223	435.260	563.319	678.346	791.430	905.473	976.510	1073.563	1160.595	1275.622	1461.701	1576.728	1663.760	1819.861
b-18	54.034	167.118	264.171	363.239	434.276	562.335	677.362	790.446	904.489	975.526	1072.579	1159.611	1274.638	1460.717	1575.744	1662.776	1818.877
b+18	90.055	203.139	300.192	399.260	470.297	598.356	713.383	826.467	940.510	1011.547	1108.600	1195.632	1310.659	1496.738	1611.765	1698.797	1854.898
y	175.119	262.151	377.178	563.257	678.284	765.316	862.369	933.406	1047.449	1160.533	1275.560	1403.619	1474.656	1573.724	1670.777	1783.861	1854.898
y-17	158.116	245.148	360.175	546.254	661.281	748.313	845.366	916.403	1030.446	1143.530	1258.557	1386.616	1457.653	1556.721	1653.774	1766.858	1837.895
i	44.049	86.096	70.065	72.081	44.049	101.071	88.039	86.096	87.055	44.049	70.065	60.044	88.039	159.092	88.039	60.044	129.113
	Arg	Ser	Asp	Trp	Asp	Ser	Pro	Ala	Asn	Leu	Asp	Gln	Ala	Val	Pro	Ile	Ala

Figure S6. MALDI-TOF-MS/MS spectra and identification results of OPN peptides: top) *m/z* 965.5, bottom) *m/z* 1854.9, from 5 µg/mL rhOPN digest using TET microchip at flow rate of 300 µL/h.

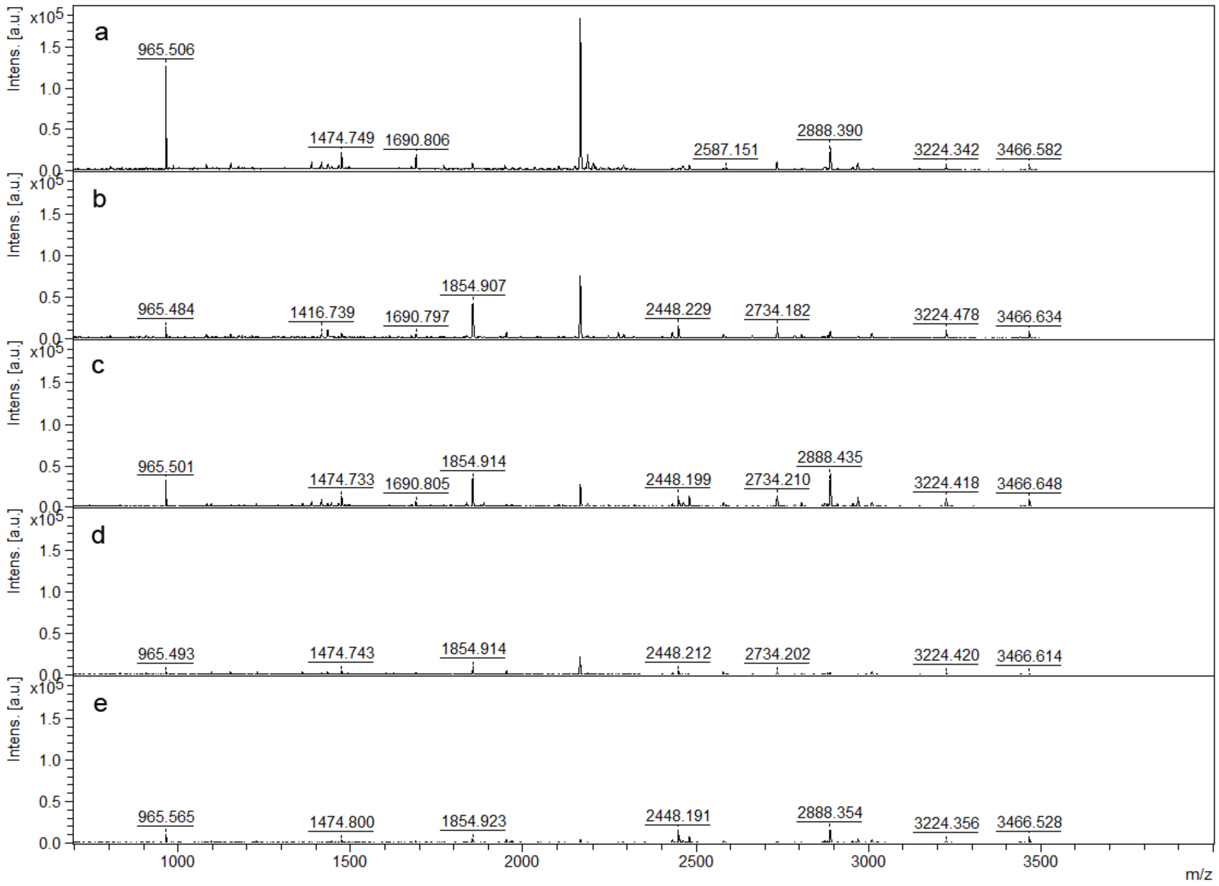

Figure S7. MALDI-TOF-MS spectra of 5 µg/mL rhOPN digests obtained from five consecutive runs using the same TET chip with flow rate 600 µL/h. a) run 1, b) run 2, c) run 3, d) run 4 and e) run 5. All peaks with labels are OPN digest peptides.

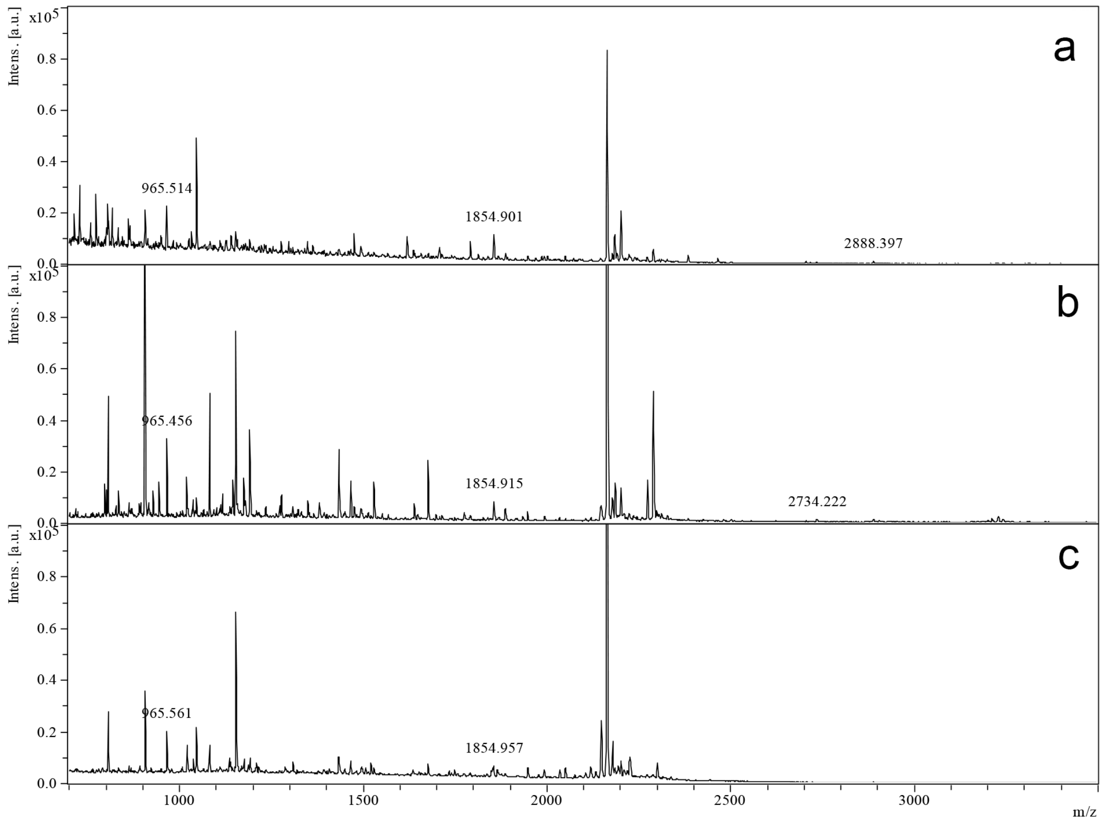

Figure S8. MALDI-TOF-MS spectra of 100 ng/mL rhOPN digests obtained from three different TET chips (a, b, and c) with a flow rate of 300 μL/h.

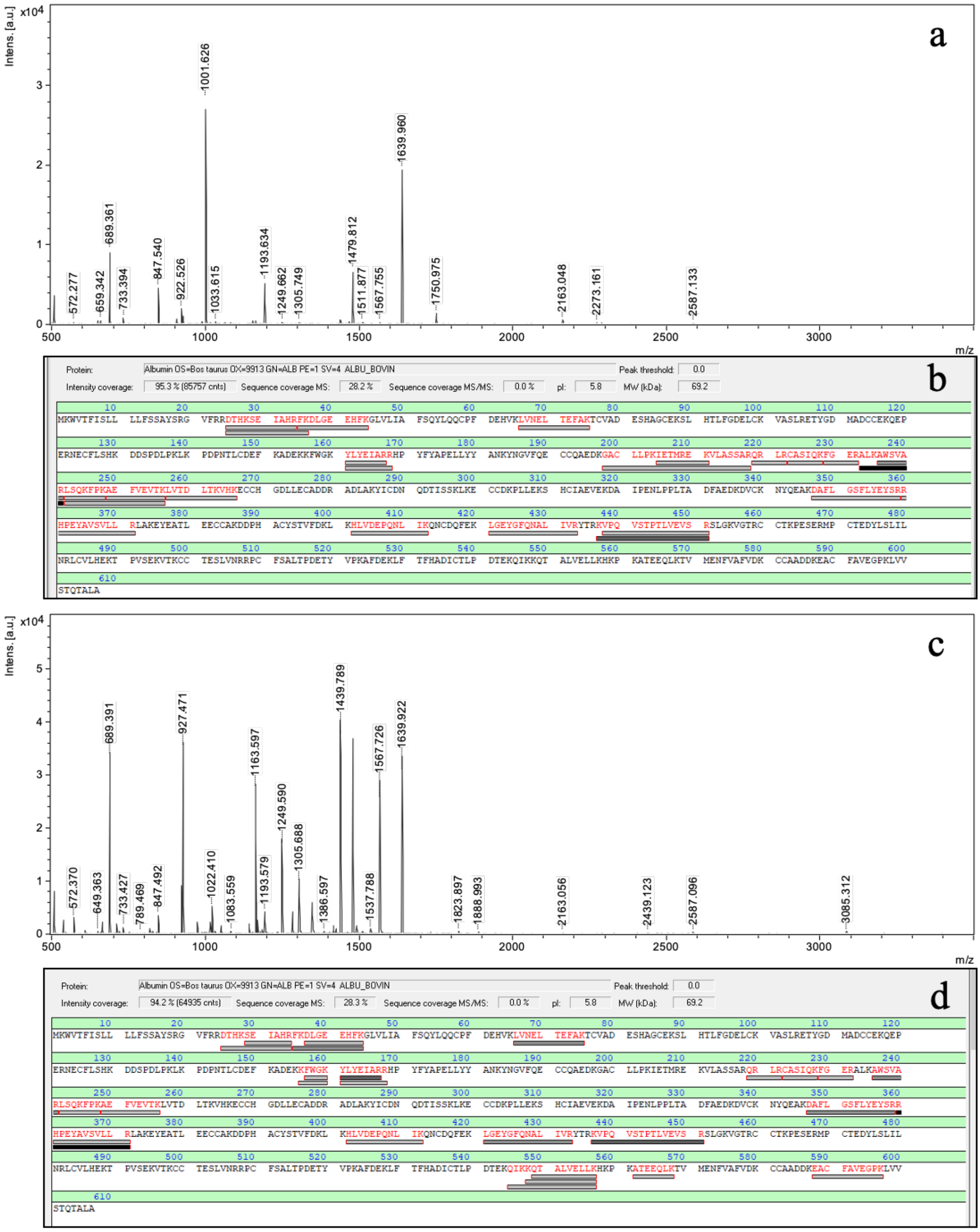

Figure S9. MALDI-TOF-MS spectra of 100 µg/mL BSA digests obtained from a) TET chip and c) conventional TD. b) and d) are identification results for BSA TET chip digest and BSA conventional TD digest, respectively.

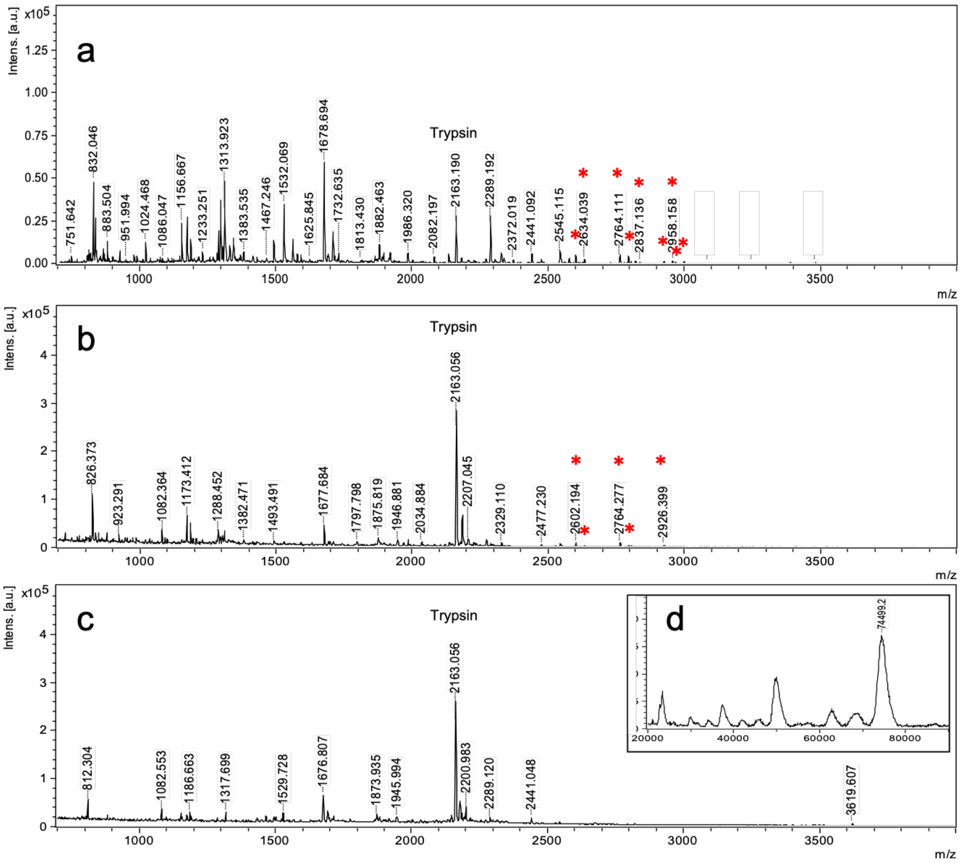

Figure S10. MALDI-TOF-MS spectra of IgG samples. a) 100 µg/mL IgG digest using conv. TD. b, c and d) 100 µg/mL IgG digest using different TET microchips. c and d) are from the same chip but different detection ranges. Glycopeptides are marked with red stars.

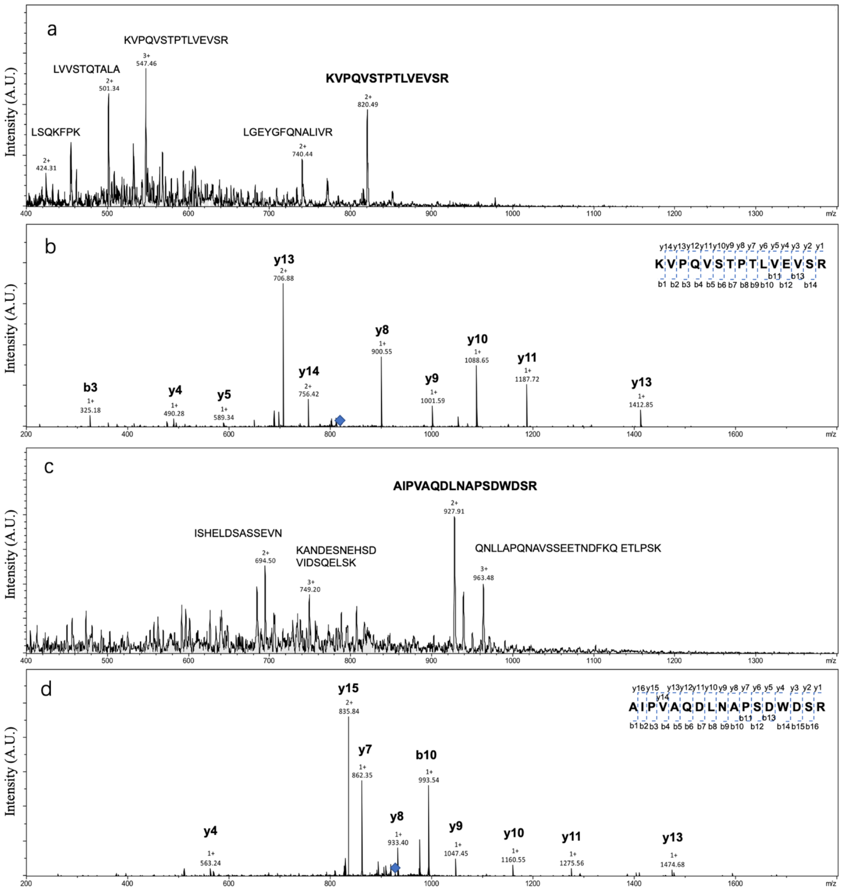

Figure S11. ESI-MS and ESI-MS/MS spectra of protein BSA and OPN digests with online TET microchip digestion and MS detection. a) ESI-MS of 100 µg/mL BSA online digest, b) ESI-MS/MS of parent ion of *m/z* 820 (2+, marked with blue diamond) from BSA online digest, c) ESI-MS of 100 µg/mL rhOPN online digest, d) ESI-MS/MS of parent ion of m/z 928 (2+, marked with blue diamond) from rhOPN online digest.

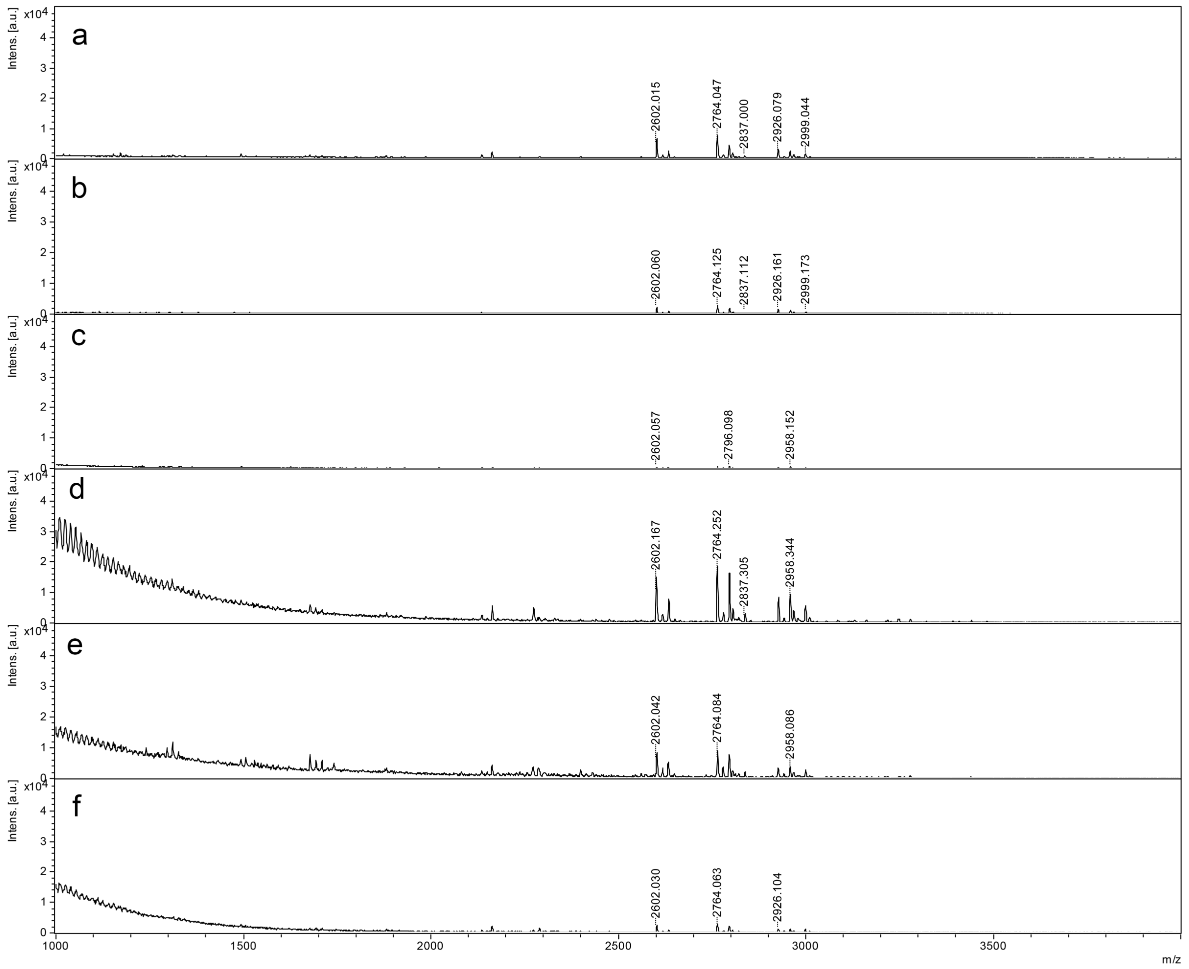

Figure S12. MALDI-TOF-MS spectra of the elution fraction after glycopeptide enrichment using TEA chips, 2 µg IgG digest with a loading solution of 86% ACN/0.1%TFA (v/v) in H_2O. a-c) run 1, 2 and 3 at flow rate of 300 µL/h. d-f) run 1, 2 and 3 at flow rate of 600 µL/h. Glycopeptides are found in the range of 2200 to 4000 Da.

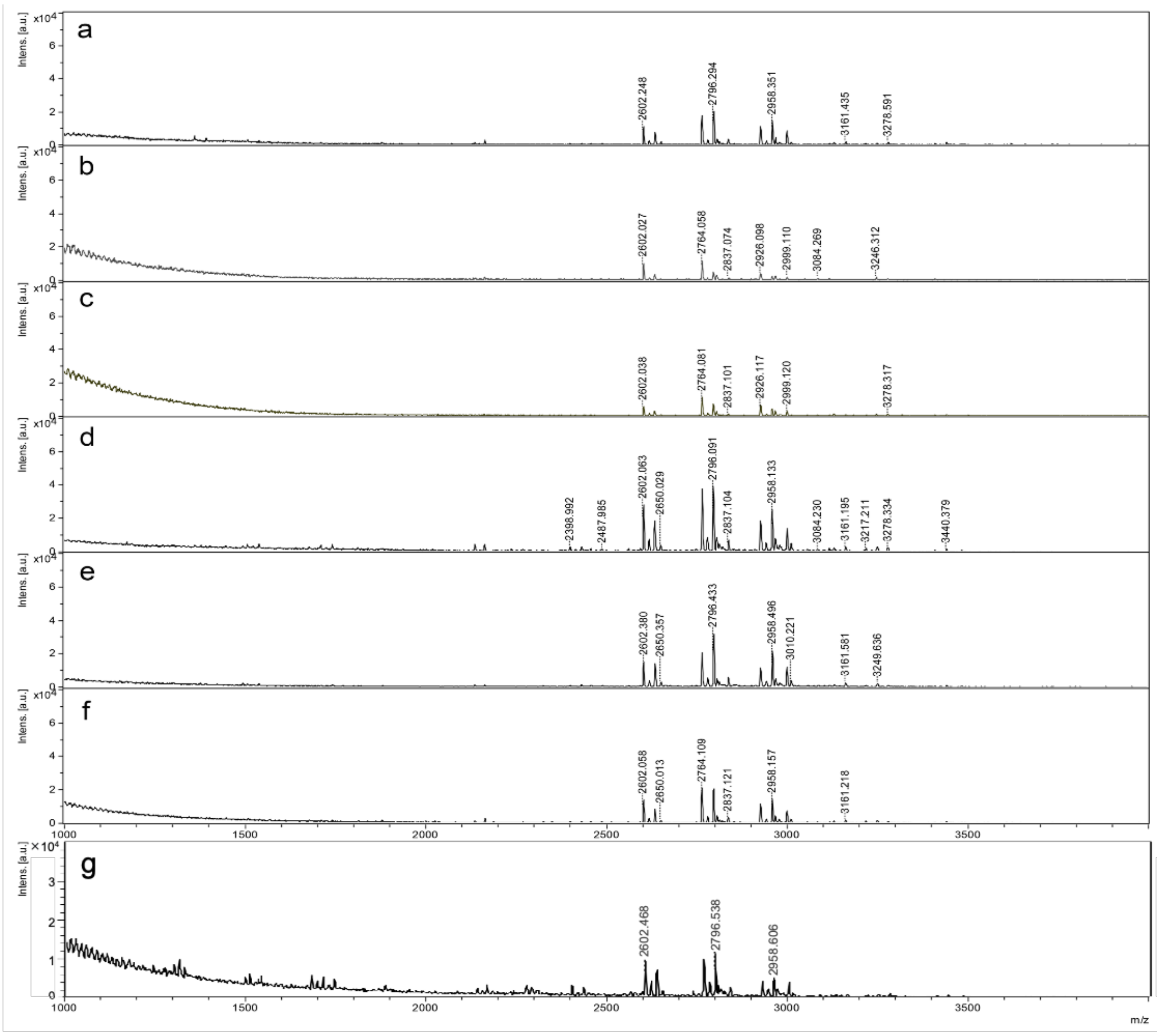

Figure S13 a-g). MALDI-TOF-MS spectra of the elution fraction after glycopeptide enrichment using TEA chips, 2 µg IgG digest with loading solution of 86% ACN/1%TFA (v/v) in H_2O. a-c) run 1, 2 and 3 at flow rate of 300 µL/h. d-g) run 1, 2, 3 and 4 at flow rate of 600 µL/h. Glycopeptides are found in the range of *m/z* 2200 to 4000 .

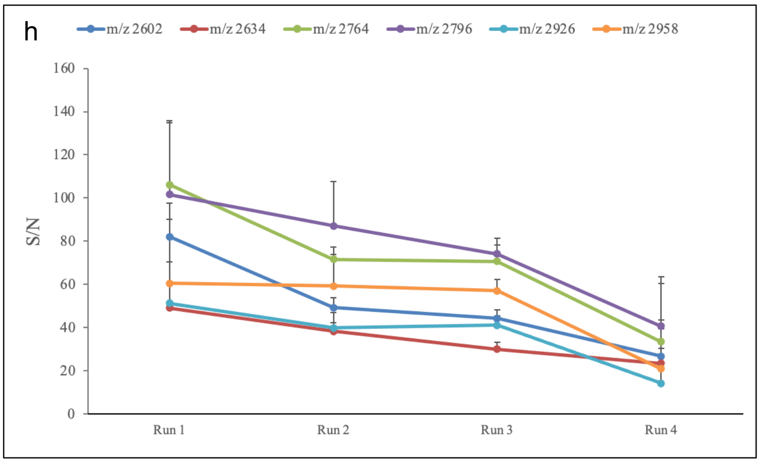

Figure S14 h). The average S/N values of the 6 most abundant glycopeptides in 4 consecutive runs after glycopeptide enrichment from 2 µg IgG digest using TEA microchips at flow rate of 600 µL/h. 5 spots on the MALDI plate were analyzed for each sample.

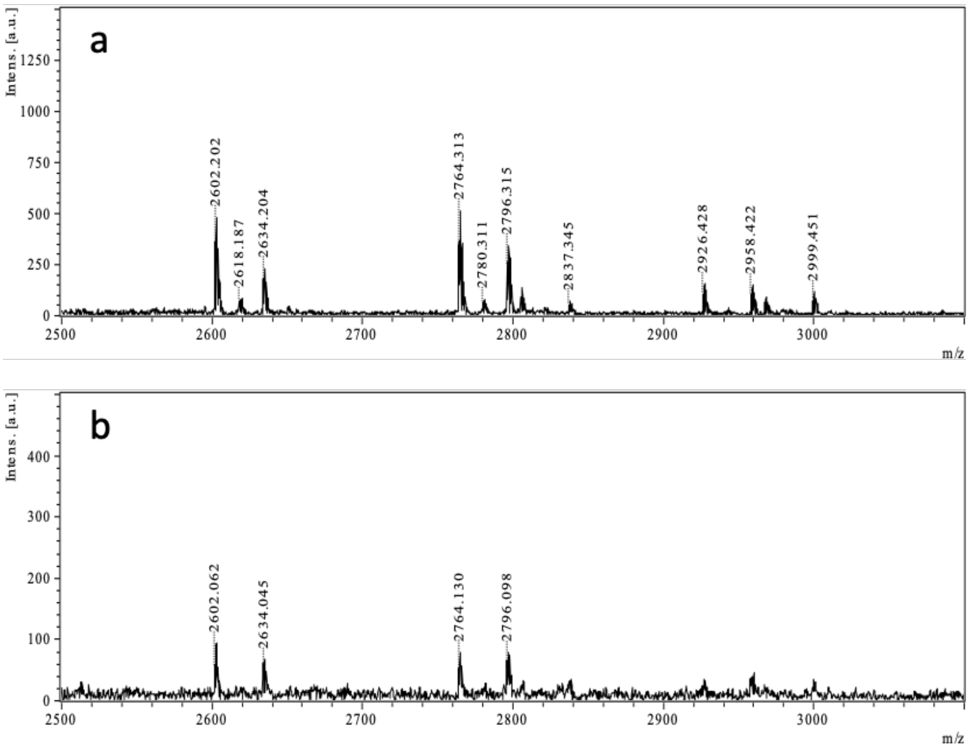

Figure S14 MALDI-TOF-MS spectra of detected IgG glycopeptides using TEA chips with different loading concentrations of IgG digest, a) 40 µg/ml, b) 10 µg/ml. Runs were carried out at flow rate of 600 µL/h. Loading solution: 86% ACN/1%TFA (v/v) in water.

Table S2. Glycan composition and structure of IgG glycopeptides after enrichment using TEA chips or commercial HILIC tips. Detected glycopeptides are marked with √. Blue square: N-Acetylglucosamine (NAc). red triangle: fucose (Fuc). green circle: manose (Hex). yellow circle: galactose (Hex). purple diamond: sialic acid (Sia).

No.	Theoretical m/z	TEA chip	HILIC	Glycan composition	Glycan structure	Amino acid sequence
1	2398.977	√	√	(Hex)3 (NAc)3 (Fuc)1	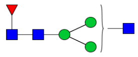	EEQFNSTFR (IgG2)
2	2430.967	√	√	(Hex)3 (NAc)3 (Fuc)1	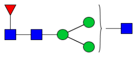	EEQYNSTYR (IgG1)
3	2455.998	√		(Hex)3 (NAc)4	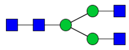	EEQFNSTFR (IgG2)
4	2487.988	√	√	(Hex)3 (NAc)4	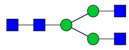	EEQYNSTYR (IgG1)
5	2561.029	√	√	(Hex)4 (NAc)3 (Fuc)1	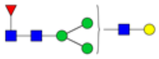	EEQFNSTFR (IgG2)
6	2593.019	√	√	(Hex)4 (NAc)3 (Fuc)1	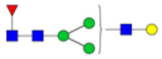	EEQYNSTYR (IgG1)
7	2602.056	√	√	(Hex)3 (NAc)4 (Fuc)1	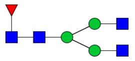	EEQFNSTFR (IgG2)
8	2618.051	√	√	(Hex)4 (NAc)4	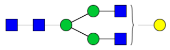	EEQFNSTFR (IgG2)
9	2634.046	√	√	(Hex)3 (NAc)4 (Fuc)1	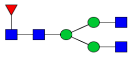	EEQYNSTYR (IgG1)
10	2650.041	√	√	(Hex)4 (NAc)4	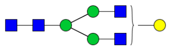	EEQYNSTYR (IgG1)
11	2764.109	√	√	(Hex)4 (NAc)4 (Fuc)1	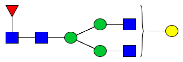	EEQFNSTFR (IgG2)
12	2780.104	√	√	(Hex)5 (NAc)4	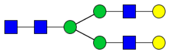	EEQFNSTFR (IgG2)
13	2796.099	√	√	(Hex)4 (NAc)4 (Fuc)1	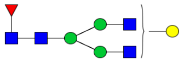	EEQYNSTYR (IgG1)
14	2805.135	√	√	(Hex)3 (NAc)5 (Fuc)1	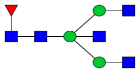	EEQFNSTFR (IgG2)
15	2812.094	√	√	(Hex)5 (NAc)4	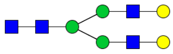	EEQYNSTYR (IgG1)
16	2821.13	√	√	(Hex)4 (NAc)5	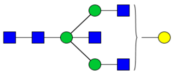	EEQFNSTFR (IgG2)
17	2837.125	√	√	(Hex)3 (NAc)5 (Fuc)1	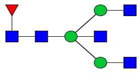	EEQYNSTYR (IgG1)
18	2926.162	√	√	(Hex)5 (NAc)4 (Fuc)1	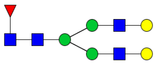	EEQFNSTFR (IgG2)

19	2941.136	√	√	(Hex)4 (NAc)4 (Sia)1	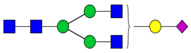	EEQYNSTYR (IgG1)
20	2958.152	√	√	(Hex)5 (NAc)4 (Fuc)1	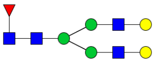	EEQYNSTYR (IgG1)
21	2967.188	√	√	(Hex)4 (NAc)5 (Fuc)1	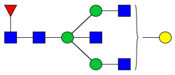	EEQFNSTFR (IgG2)
22	2999.178	√	√	(Hex)4 (NAc)5 (Fuc)1	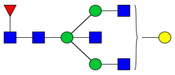	EEQYNSTYR (IgG1)
23	3084.353	√	√	(Hex)3 (NAc)4 (Fuc)1	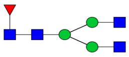	TKPREEQFNSTFR (IgG2)
24	3129.241	√	√	(Hex)5 (NAc)5 (Fuc)1	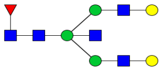	EEQFNSTFR (IgG2)
25	3161.231	√	√	(Hex)5 (NAc)5 (Fuc)1	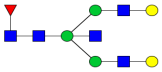	EEQYNSTYR (IgG1)
26	3217.257	√	√	(Hex)5 (NAc)4 (Fuc)1 (Sia)1	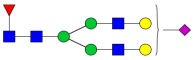	EEQFNSTFR (IgG2)
27	3246.406	√	√	(Hex)4 (NAc)4 (Fuc)1	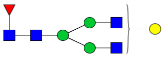	TKPREEQFNSTFR (IgG2)
28	3249.247	√	√	(Hex)5 (NAc)4 (Fuc)1 (Sia)1	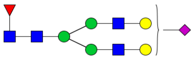	EEQYNSTYR (IgG1)
29	3278.396	√	√	(Hex)4 (NAc)4 (Fuc)1	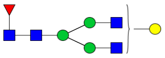	TKPREEQYNSTY R (IgG1)
30	3408.459	√	√	(Hex)5 (NAc)4 (Fuc)1	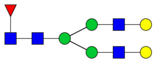	TKPREEQYNSTFR (IgG2)
31	3440.449	√	√	(Hex)5 (NAc)4 (Fuc)1	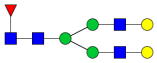	TKPREEQYNSTY R (IgG1)

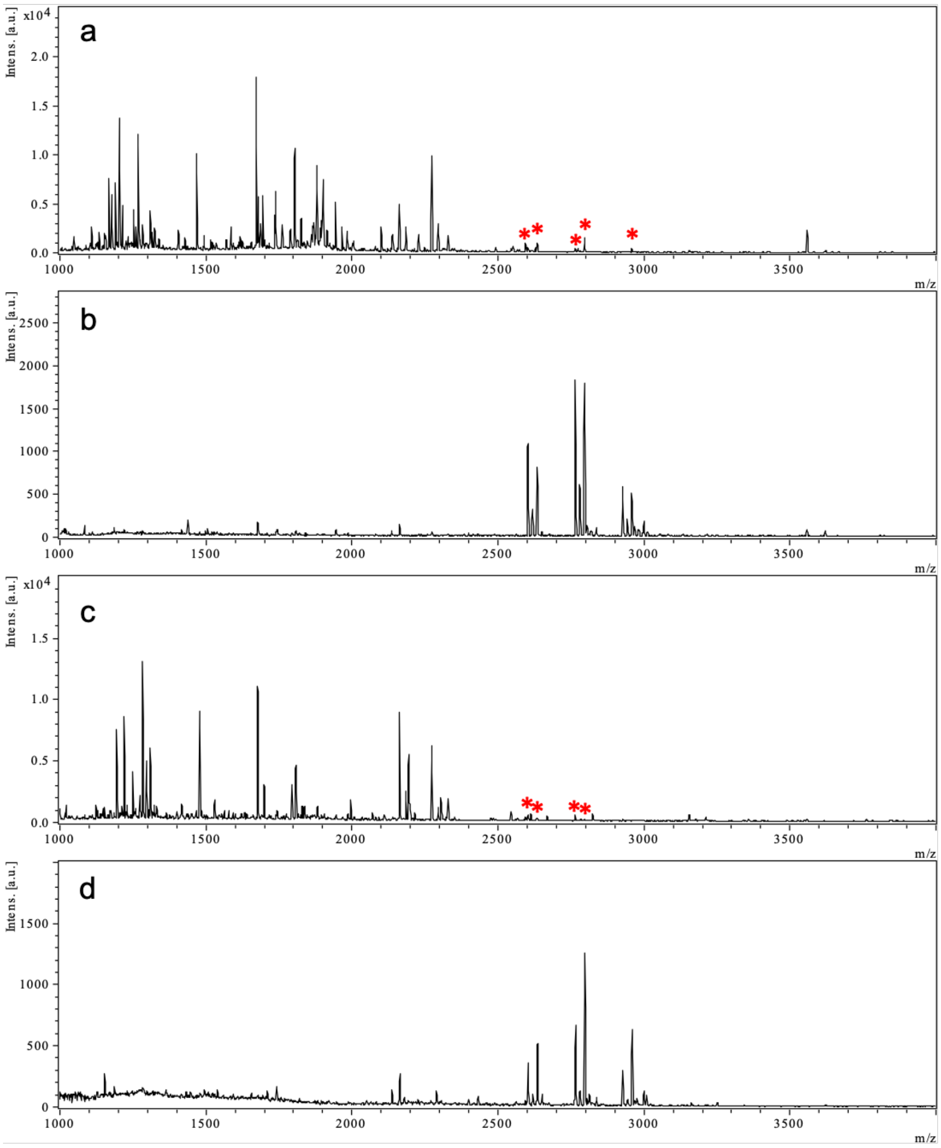

Figure S15. MALDI-TOF-MS spectra of a) healthy human serum IgG digest and b) enriched IgG glycopeptides from healthy human serum IgG digest, c) CoV serum IgG digest and d) enriched IgG glycopeptides from CoV human serum IgG digest. Red stars in a) and c) mark glycopeptides in IgG digests without enrichment. In b) and d), peaks located in the range of *m/z* 2300 to 4000 are IgG glycopeptides.
